# Biodegradable plastic formulated from chitosan of *Aristeus antennatus* shells with castor oil as a plasticizer agent and starch as a filling substrate

**DOI:** 10.1038/s41598-024-61377-9

**Published:** 2024-05-15

**Authors:** Ayaat R. El Feky, Mohammed Ismaiel, Murat Yılmaz, Fedekar M. Madkour, Ahmed El Nemr, Hassan A. H. Ibrahim

**Affiliations:** 1 Oceanographic Sciences Department, Faculty of Science, Port Said University, Port Fuad, Egypt; 2 Bahçe Vocational School, Department of Chemistry and Chemical Processing Technologies, Osmaniye Korkut Ata University, Osmaniye, 80000 Turkey; 3 Environment Division, National Institute of Oceanography and Fisheries (NIOF), Kayet Bey, Elanfoushy, Alexandria Egypt

**Keywords:** *Aristeus antennatus*, Deacetylation, Biofilms, Chitosan, Castor oil, Chitin, Bioplastic, Environmental chemistry, Chemical engineering

## Abstract

Biodegradable plastics are those subjected easily to a degradation process, in which they can be decomposed after disposal in the environment through microbial activity. 30 bioplastic film formulations based only on chitosan film were used in the current investigation as a positive control together with chitosan film recovered from chitin-waste of locally obtained *Aristeus antennatus*. Additionally, castor oil was used as a plasticizer. While the yield of chitosan was 18% with 7.65% moisture content and 32.27% ash in the shells, the isolated chitin had a degree of deacetylation (DD) of 86%. The synthesized bioplastic films were characterized via numerous criteria. Firstly, the swelling capacity of these biofilms recorded relatively high percentages compared to polypropylene as synthetic plastic. Noticeably, the FTIR profiles, besides DSC, TGA, and XRD, confirmed the acceptable characteristics of these biofilms. In addition, their SEM illustrated the homogeneity and continuity with a few straps of the chitosan film and showed the homogeneous mixes of chitosan and castor oil with 5 and 20%. Moreover, data detected the antibacterial activity of different bioplastic formulas against some common bacterial pathogens (*Enterococcus feacalis*, *Kelbsiella pnumina*, *Bacillus subtilis*, and *Pseudomonas aeruginosa*). Amazingly, our bioplastic films have conducted potent antimicrobial activities. So, they may be promising in such a direction. Further, the biodegradability efficacy of bioplastic films formed was proved in numerous environments for several weeks of incubation. However, all bioplastic films decreased in their weights and changed in their colors, while polypropylene, was very constant all the time. The current findings suggest that our biofilms may be promising for many applications, especially in the field of food package protecting the food, and preventing microbial contamination, consequently, it may help in extending the shelf life of products.

## Introduction

Every year, more and more petroleum-based plastic trash has accumulated, reaching billions of tons of untreated, undegraded plastics that contribute significantly to environmental degradation. Many plastics can absorb natural and mineral toxins from the concentrate and the environment up to a million times the concentrations found in seawater^1,2^. In addition, the primary sources of chitin are the shells of marine crustaceans, which are mostly made up of 20–30% chitin, 30–50% calcium carbonate, 30–40% protein and calcium phosphate^3,4^. A variety of industries, including agriculture, wastewater treatment, medication delivery, tissue engineering, molecular imprinting, cosmetics, and food preservation, have been reported by numerous authors to use chitin and its derivatives^5–9^. Commercially, the crustacean industry produces vast amounts of waste that are thrown out every day and poses a disposal issue. This abundant waste material can be used more effectively for commercial purposes by being transformed into forms that have added value, such as nutrients and other useful biochemical substances like chitin and chitosan^10–13^.

Interestingly, the commercialization of an alternative technique for creating biodegradable polymers from sustainable and renewable resources is necessary to address the problem of environmental contamination^14^. The biodegradation products of the biofilms are not generally harmful and can be ingested by other living things^15^. More specifically, biodegradable biofilms are polymers that, through the enzymatic action of particular bacteria, are mineralized into methane, inorganic chemicals, water, carbon dioxide, or biomass. As a result, they might be a good and sustainable alternative to traditional petrochemical plastics^16,17^.

Foodborne infections are thought to be responsible for 48 million illnesses, 128,000 hospital admissions, and 3,000 fatalities per year in the United States, according to the Centers for Disease Control and Prevention (CDC). Therefore, maintaining the products' nutritional and organoleptic qualities while assuring their microbiological safety is still a top priority today^18^. Antimicrobial packaging can also be used to expand the margin of safety and quality. This packaging may stop microbes from growing on the product's surface, extending the product's shelf life^19,20^.

Within the center of a few sorts of characteristic polymers, chitosan, a polysaccharide determined from chitin, has picked up a reputation in a few ranges, from biopharmaceutical and biomedical applications to squander water treatment and nourishment industry, because of its multifunctional properties^21^. In such a manner, chitosan has long potential as a characteristic and biodegradable substitute for petroleum-based plastics in a few applications^22–24^.

Surprisingly, the thermo-mechanical plasticization of chitosan can be an intriguing substitute for the traditional casting technique and enables the production of chitosan movies on a larger scaleThermo-mechanical medications in an inner blender within the nearness of water, acidic corrosive and distinctive polyols^25^. A one-step expulsion handle within the nearness of glycerol and acidic corrosive arrangement has been detailed for chitosan plasticization^26^. Additionally, thermo-compression and melt-mixing techniques were used to create thermoplastic starch/chitosan films^27^, dissolve expulsion^28^ or blown expulsion^29^. However, a biodegradable product can decompose into natural raw materials and disappear into nature using safe, dependable, and relatively rapid biological processes Biofilms can also be made utilizing microscopic organisms and some of the time different nanometer-sized particles generally polysaccharides^30^.

Therefore, the present study was suggested mainly to develop an eco-friendly biodegradable plastic material to be used in the future as an alternative to synthetic plastic. It also seeks to address the potential of the by-products of the Egyptian marine fisheries like crustacean wastes as a valuable source of biomaterials and natural compounds. Collectively, the current investigation aims to extract chitosan from shrimp wastes besides using castor as a plasticizer agent and starch as a filling substrate. Moreover, the chemical, physical and biological characteristics of the bioplastic films formulated will be completely evaluated.

## Material and methods

### Bacterial reference strains and media used

The bacteria used as reference strains were; Gram-negative bacteria such as *Enterococcus feacalis* and *Kelbsiella pnumina* and Gram-positive bacteria, such as *Bacillus subtilus* and *Pseudomonas aeruginosa.* Kindly, they were provided from the Microbiology Lab, at NIOF and maintained on nutrient agar slants and then kept at 4 °C. Additionally, the medium for antimicrobial activity was nutrient broth (NB) medium, which was composed of (g/L): beef extract, 1; peptone, 5; yeast extract, 2; sodium chloride, 5. Agar (15–20) was added to obtain nutrient agar medium^31^.

### Preparation of shrimp samples wastes

Shells of the pink shrimp were collected from a local fish market at Port Said, Egypt, to be used as substrates for preparing sample wastes to extract chitin and then via the deacetylation process to chitosan. When shrimp processing waste from *A. antennatus* flesh portions was discarded, shell components, including a complete cephalothorax and abdominal exoskeleton, were collected. It was identified with the aid of the Marine Science Department, Faculty of Science, Port Said University, Egypt. Before and during transport to the laboratory, representative samples of shrimp waste were selected, sealed in plastic bags and stored at –20 °C. According to Mohammed et al.^32^, they were cleaned with purified water, allowed to air dry, and then baked for four days straight at 60 °C.

### Extraction of chitosan

The chitin extraction was prepared according to the method of Mohammed et al.^32^ by using a combination of three major procedures. First, 10 kg of discarded shrimp shells were treated with 4% NaOH for 24 h at room temperature. When the pH became neutral, the alkali was removed from the shells and repeatedly rinsed with distilled water. Shells that had been demineralized by 4% HCl at room temperature for 12 h to produce chitin underwent deproteinization as a result of this process. Chitin had its acid removed, washed with distilled water, and then allowed to finish drying at room temperature. Repeating this procedure using 1% HCl and 2% NaOH. The obtained chitin was still slightly pink. Chitin was then treated with 1% potassium permanganate and 1% oxalic acid for an additional 30 to 2 h to further decolorize it^33^.

Finally, to create chitosan, the decolorized chitin underwent a deacetylation procedure by being exposed to 70% (w/v) NaOH for three days at 30 °C. According to Premasudha et al.^34^, the alkali fraction found in chitosan was separated by centrifugation at 4000 rpm for 15 min, excess alkali was drained off, and additional washing with distilled water was carried out until pH reached neutral. To facilitate further research, the resulting chitosan fraction was subsequently dried at 40 °C overnight.

### Physiochemical characteristics of extracted chitosan

#### Determination of chitosan yield

The ratio of the dry weight of chitosan flakes to the wet weight of shrimp shell, as determined by Eq. (1), was used to calculate the chitosan yield (%)^35^:

1 $$Chitosan Extraction Yields \%= \frac{ Extracted chitosan (g)}{Shrimp shell waste (g)} \times 100$$

#### Degree of deacetylation (DD%)

The acid–base titration method through some modifications was applied to determine the degree of deacetylation (DDA) of the derived chitosan experimentally^36^. Briefly stated, 0.125 g of derived chitosan was weighed, mixed in 30 mL of 0.1 M standard HCl aqueous solution, around 5 drops of methyl orange were added as an indicator, and then vigorously stirred for 30 min until the chitosan-HCl mixture was homogenous at room temperature. It was then titrated with 0.1 M NaOH solution until the red chitosan solution turned orange. Using Eq. (2), the chitosan's percentage degree of deacetylation (DD%) was determined.

2 $$DD \% = \frac{C1V1-C2V2}{M \times 0.0994} \times 0.016 \times 100$$

where; *C*_*1*_ and *C*_*2*_ are the mol/L concentrations of standard HCl and NaOH aqueous solutions, *V*_*1*_ is the volume of 0.1 M HCl solution consumed during titration (mL), *V*_*2*_ is the volume of 0.1 M NaOH solution ingested during titration (mL), and *M* is the weight of derived chitosan (g). The weight of the NH_2_ group in 1 mL of standard 1 M HCl solution, expressed in grams, is a standard factor in Eq. (2), and the direct proportional ratio of the NH_2_ group by weight in the resulting chitosan is 0.0994^33^.

#### Moisture content

Moisture content and residue on ignition were determined using the method described by Kumari et al.^37^. The gravimetric method was used to determine the loss on drying of the synthesized chitosan. The mass loss of the water was calculated by drying the sample to a consistent weight and weighing it both before and after drying. According to Eq. (3), the water mass (or weight) represented the difference between the wet and oven-dry sample weights.

3 $$Moisture Content= \frac{Wet weight-Dry weight}{Dry weight} \times 100$$

#### Solubility of chitosan

One gram of chitosan was dissolved in 99 ml of distilled water with 1% glacial acetic acid using a magnetic stirrer for 30 min. The sample was extracted, the residue was filtered out using Whatman No. 1 filter paper, and then the sample was weighed, according to Kumari et al.^37^.

### Bioplastic film synthesis

#### Formulation and production of bioplastic film composites

The soluble chitosan in glacial acetic acid (pH 4) was prepared by dissolving 1 g of chitosan in 99 mL of 1% glacial acetic acid. The corn starch was also prepared by dissolving 5 g of starch in 100 mL of distilled water while stirring on a hotplate at 50 °C for 20 min. The gelatinized chitosan and corn starch solutions were mixed well and then mixed again with various concentrations of castor oil as a plasticizer to increase flexibility with constant agitation. To create a fine solution, homogenization of the chitosan/starch/castor mixture was carried out five times. Afterwards, each biofilm sample is poured into the strangle mold casts and then allowed to be dried overnight at 60 °C in an oven to obtain the films. Finally, all the films were conditioned in a desiccator and left for at least 48 h before analysis^38^. A base of 100 will be used to formulate the chitosan/starch/oil composition, as shown in Table 1. All experimental work was repeated three times and mean values were reported with standard deviation.

**Table 1:** Different formulations of biofilm synthesized based on chitosan extracted from chitin-wastes of locally collected *A. antennatus.*

Cast film	Polymer suspension (%)		Plasticizer (%)		
	Chitosan	Starch	Glycerol	Castor	Paraffin
Formulation I					
1G	10	45	45	–	–
2G	30	35	35	–	–
3G	50	25	25	–	–
4G	70	15	15	–	–
5G	75	10	15	–	–
6G	78	5	18	–	–
7G	70	10	20	–	–
8G	85	5	10	–	–
9G	90	5	5	–	–
10G	97	2	1	–	–
Formulation II					
1C	10	45	–	45	–
2C	30	35	–	35	–
3C	50	25	–	25	–
4C	70	15	–	15	–
5C	75	10	–	15	–
6C	78	5	–	18	–
7C	70	10	–	20	–
8C	85	5	–	10	–
9C	90	5	–	5	–
10C	97	2	–	1	–
Formulation III					
1P	10	45	–	–	45
2P	30	35	–	–	35
3P	50	25	–	–	25
4P	70	15	–	–	15
5P	75	10	–	–	15
6P	78	5	–	–	18
7P	70	10	–	–	20
8P	85	5	–	–	10
9P	90	5	–	–	5
10P	97	2	–	–	1
Positive control					
PC	100	–	–	–	–

*G Glycerol, *C* Castor, *P* Paraffin, *PC* Positive control (chitosan only).

### Characterization of chitosan and synthesized bioplastic films

#### Fourier transform infrared (FTsIR) analysis of chitosan

Before being dried for the FTIR analysis, the specimens were washed in water for 4 min to remove any unreacted acid. El-Zahhar et al.^39^ report employing FTIR in the attenuated reflection (ATR) mode with 32 scans at a resolution of 4 cm^−1^ in the wavenumber range of 4000 and 600 cm^−1^ to observe the functional groups on the surface of the materials.

#### Degree of swelling

The amount of water taken in (*WU*) or the degree of swelling of the membranes was measured. A previously dried membrane sample (*Wd*) was weighed and immersed in room-temperature distilled water. After 24 h, the sample was removed from the aqueous medium, lightly dried to eliminate excess water, and reweighed (*Ww*). The following Eq. (4) was used to calculate the degree of swelling^40^:

4 $$WU= \frac{Ww-Wd}{Wd} \times 100$$

#### Tensile strength

The mechanical properties of biofilms in terms of tensile strength and elongation % were measured by Universal Testing Machine Tinius Olsen model HTND-1,5 kVA (Shimadzu, Japan). The films with a dimension of 7 × 2 cm with a speed of 50 mm/min and 10 N load cell. An equipment called a thickness gauge was used to measure the sample thickness (Mitutoyo, Japan).

However, tensile strength was assessed by lengthening the sample and measuring the weight it could support before breaking. According to the following Eqs. (5), (6) and (7), the tensile strength was estimated by dividing the highest load by the sample's initial cross-sectional area^41^.

5 $$Tensile strength \left(MPa\right)= \frac{F \left(Kg\right)}{A \left({cm}^{2 }\right)} \times 100$$

where; *F* was measured load before breaking (Kg), while *A* was a cross-sectional area (cm^2^) of the sample (width × thickness).

6 $$\mathrm{Elongation of break }(\mathrm{\%}) =\frac{The increase in length of breaking point (mm)}{Original length} \times 100$$

7 $$E = \frac{\partial }{\mathcal{E}}$$

where; *E* is Young's modulus, pressure units, *ε* = strain, or proportional deformation (change in length divided by original length), dimensionless and ∂ = uniaxial stress, or uniaxial force per unit surface, pressure units.

#### Thermogravimetric analysis (TGA) and differential scanning calorimetry (DSC)

The resistance of the film to the thermal contact influence allowed for the examination of thermal properties. Thermal properties analysis was measured by the DSC-TGA by a thermogravimetric analyzer (TERIOS Universal V4.5A TA Instruments). At a temperature range between 25 and 600 °C, the film was heated at a rate of 10 °C/min^38^. Weighed samples were sealed inside aluminium pans and heated at a rate of 10 °C per minute from 20 to 180 °C for 5 min before being cooled to 25 °C. Under a nitrogen flow, the measurement was carried out in a heating–cooling-heating cycle. The reference pan was an empty aluminium pan. The temperature that corresponds to the midpoint between the least and maximal values seen at the transition zone during the second heating cycle was identified as the glass transition temperature^42^.

#### X-ray diffraction analysis (XRD)

The scattering of X-rays by materials, particularly crystals, with the concomitant variation in intensity brought on by interference effects. Through the use of X-rays and the registration of the diffraction picture of the beams, it is possible to analyze the crystal structure of various materials. The XRD were obtained with a Bruker Meas Srv (D2-208219)/D2-2082019 diffractometer operating at 30 kV, 10 mA, and a Cu tube (λ = 1.54) with a 2 Theta (2*θ*) range of 10 to 80°.

#### Scanning electron microscope (SEM)

Microstructural analysis of the surface and cross sections of the synthesized biofilms were studied using an SEM technique in a JEOL JSM5410 (Japan) electron microscope. The biofilms were chopped into 5 × 1 mm^2^ pieces, which were then placed on copper stubs. A 10 kV accelerating voltage was used to view samples that had been coated with gold. For surface morphology, the images were taken at a magnification of × 10,000, and for their cross-section, at × 25,000^43^.

### Biological characterization of synthesized bioplastic films

#### Antimicrobial activity of synthesized biofilms

Initially, NB was used for detecting the antibacterial activity of the biofilms produced against different reference strains (*E. feacalis, K. pnumina*, *B. subtilis*, and *P. aeruginosa*) in liquid broth according to Cardozo et al.^44^. Seeding cultures of these strains were freshly prepared in NB with an optical density of 0.7 at an absorbance of 550 nm using a Spectronic 21D Milton Roy spectrophotometer. On the other hand, different formulas of biofilms were placed with 1 × 1 cm^2^. To achieve a positive control, 20 µL mL^–1^ of reference strains were put into test tubes containing 2 mL sterilized NB, whereas blank was used as NB without any treatments. Treatments included reference strains (20 μL mL^–1^) with different formulas of biofilm films. All tubes were incubated in a shaker (New Brunswick Scientific, Edison, N.J., U.S.A.) at 37 °C for 4 different intervals (2, 4, 12, and 24 h). The optical densities of each treatment were then measured at A550 nm, and suppression percentages were computed using Eq. (8)^45^.

8 $$ {\text{Suppression}}\;\% = \frac{{{\text{A}}_{550} \;{\text{control}} - {\text{A}}_{550} \;{\text{treatment}}}}{{{\text{A}}_{550} \;{\text{control}}}} \times 100 $$

#### Biodegradation of synthesized biofilms

Experimentally, the biodegradability tests were utilized to measure specifically biofilms, which have to be cut into small pieces and buried within various environments^46^. Briefly, the different suspensions containing microbial community as a whole were prepared from seawater (SW), marine sediment (MS), municipal wastewater (MW), garden soil (GS), and municipal house waste (HW). The bioplastic films were cut into 2 × 2 cm and put into a Petri dish. Afterwards, 20 mL of microbial solutions (SW, MS, MW, GS, and HW), which were previously diluted 5 times were added. The physical changes in biofilms such as changes in size, thickness, and color during the degradation processes were observed every 24 h^47^.

### Statistical analysis

For mechanical and antibacterial tests, all data represent the mean of triplicate results. The mean and standard deviation of the data were presented. Using the Prism 8.0.1 program, the analysis of variance (ANOVA) process was carried out with the threshold of significance set at p < 0.05. Differences were considered significant when the *p*-value was less than 0.05.

## Results and discussion

### Yield and moisture of chitosan

Chitosan yield from *A. anntenus* in this research is relatively lower where it was 18%. This was higher than that reported by Brzeski and Sauer^48^ (14% yield of chitosan from shrimp), which may be due to depolymerization of the chitosan polymer, loss of sample mass/weight from excessive removal of acetyl groups from the polymer during deacetylation, and loss of chitosan particles during washing.

It almost exactly matches William and Wid's^35^ findings, who found that the average amount of chitosan produced from the wet weight of shrimp shell waste was 19.01%. Because the natural structure of chitin (a protein) was lost during the deproteination process, less chitosan was generated when extraction was started with deproteinization. The weight of the chitosan was ultimately reduced by the prior process, which led to increased hydrolysis and losses of solid material. Moisture content in this study the shrimp shell chitosan samples had a moisture content of 7.65 ± 0.13%. The produced chitosan's moisture content was 7.77 ± 0.15%, compared to 7.50 ± 0.10% for commercial chitosan. This discovery is consistent with other studies' findings. According to Li et al.^49^, commercial chitosan can include more than 10% moisture. According to Rege et al.^50^, the moisture content of chitosan powder ranged from 7 to 11% (w/w) and had nothing to do with its DD or MW. On the other hand, Mucha et al.^51^ found that DD had a favorable impact on moisture content. The ability to absorb water will decrease as the DD increases.

### Degree of deacetylation of chitosan (DD%)

Numerous physiochemical and organic properties of chitosan, including crystallinity, hydrophilicity, degradation, and cell response, are determined by the degree of deacetylation (DD), which is usually recognized as an essential metric. According to the degree of d-glucosamine and *N*-acetyl d-glucosamine deacetylation, the biopolymer is classified as either chitin or chitosan. Various methods have been described for guaranteeing the degree of chitosan deacetylation^52^. The DD of the extracted chitosan from the current study was found to be 86%, which is very similar to Puvvada et al.^53^ DD of 89.79% and their conclusion that higher DD values were brought on by the presence of more protein, which affects the chemical, physical, and biological properties of chitosan, such as adsorption, covalent linking, and encapsulation. While Younes and Rinaudo^54^ have recorded that 70.9% and it’s the lowest value for characteristic of chitin; (DD) value depends on the raw material and the processes used for the deproteinization and the demineralization. Additionally, Muñoz et al.^55^ found that 73.6% of the chitin was deacetylated and noted that the process of DD is dependent on the type of chitin utilized as well as the time, temperature, and alkaline concentration.

### Solubility of extracted chitosan

In contrast to the 99% reported for commercial chitosan by Hossain and Iqbal^56^, the solubility from extracted chitosan in this work was 98.15%. To achieve the requisite solubility, deacetylation is thought to need to be at least 85%. Chitosan solubility increased correspondingly as the degree of deacetylation increased; samples treated with 50 and 60% NaOH yielded solubility ranging from 96.01 to 97.2%, respectively. The degree of deacetylation affects both the solubility properties of chitosan. A high degree of deacetylation appears to have higher solvency, and a degree of deacetylation appears to destitute solubility^57,58^. Moreover, the partial removal of the acetyl group and protein gives lower solubility values of chitosan solubility in inorganic acid^56^, due to the highly protonated free amino group that attracts ionic compounds^32^.

### FTIR spectrum of extracted and standard chitosan

**Figure 1:**
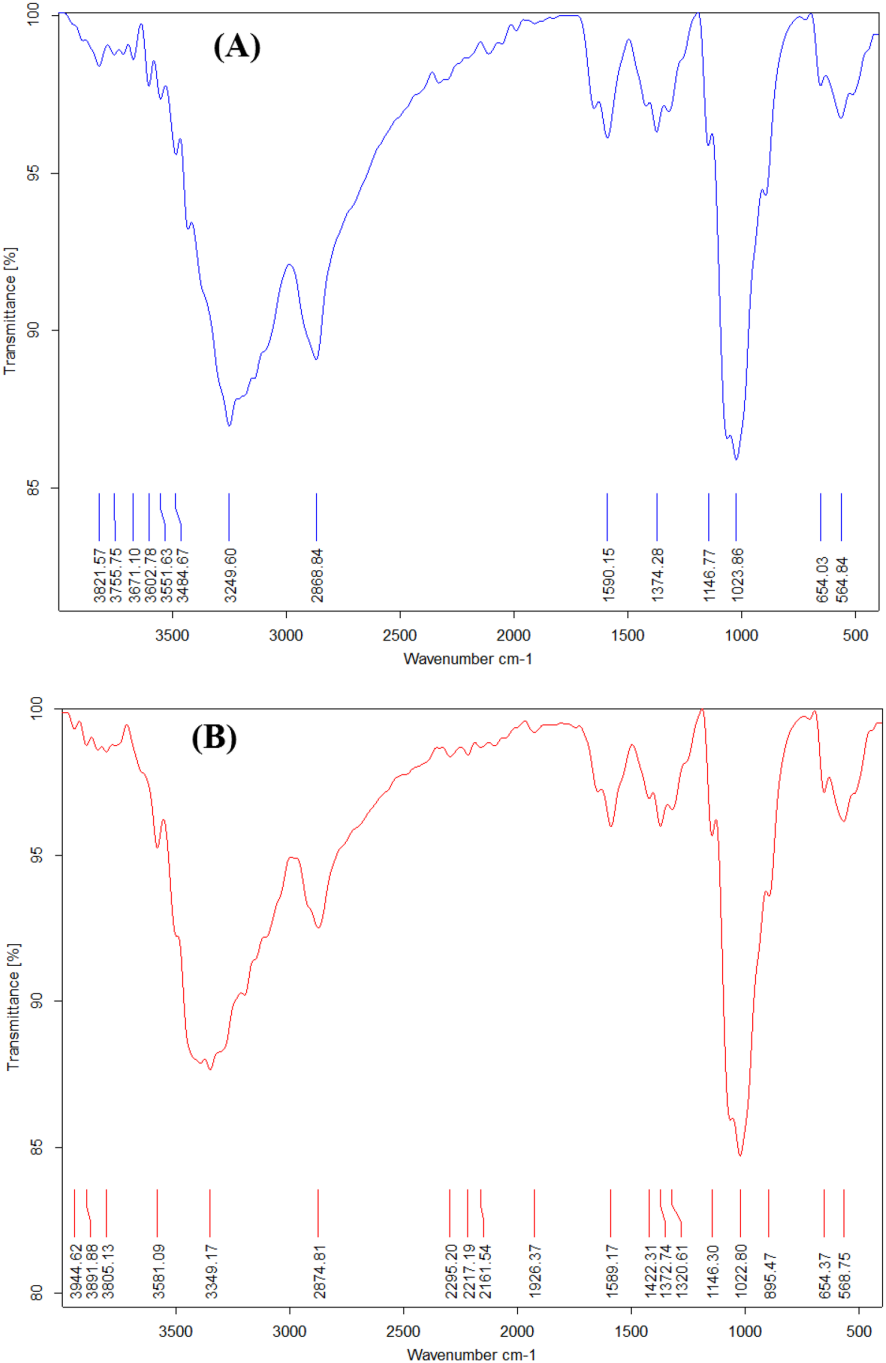
FTIR spectra of extracted chitosan (**A**) and standard chitosan (**B**).

**Figure 2:**
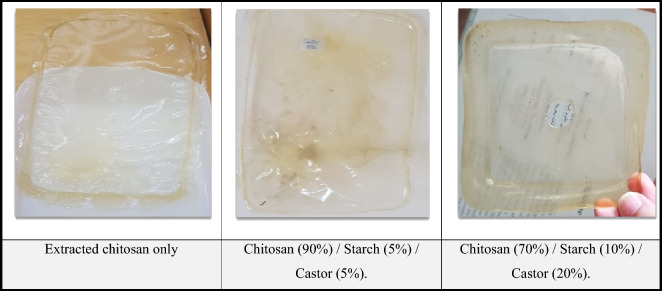
A side of the most acceptable biofilms synthesized from chitosan, starch, and castor.

**Figure 3:**
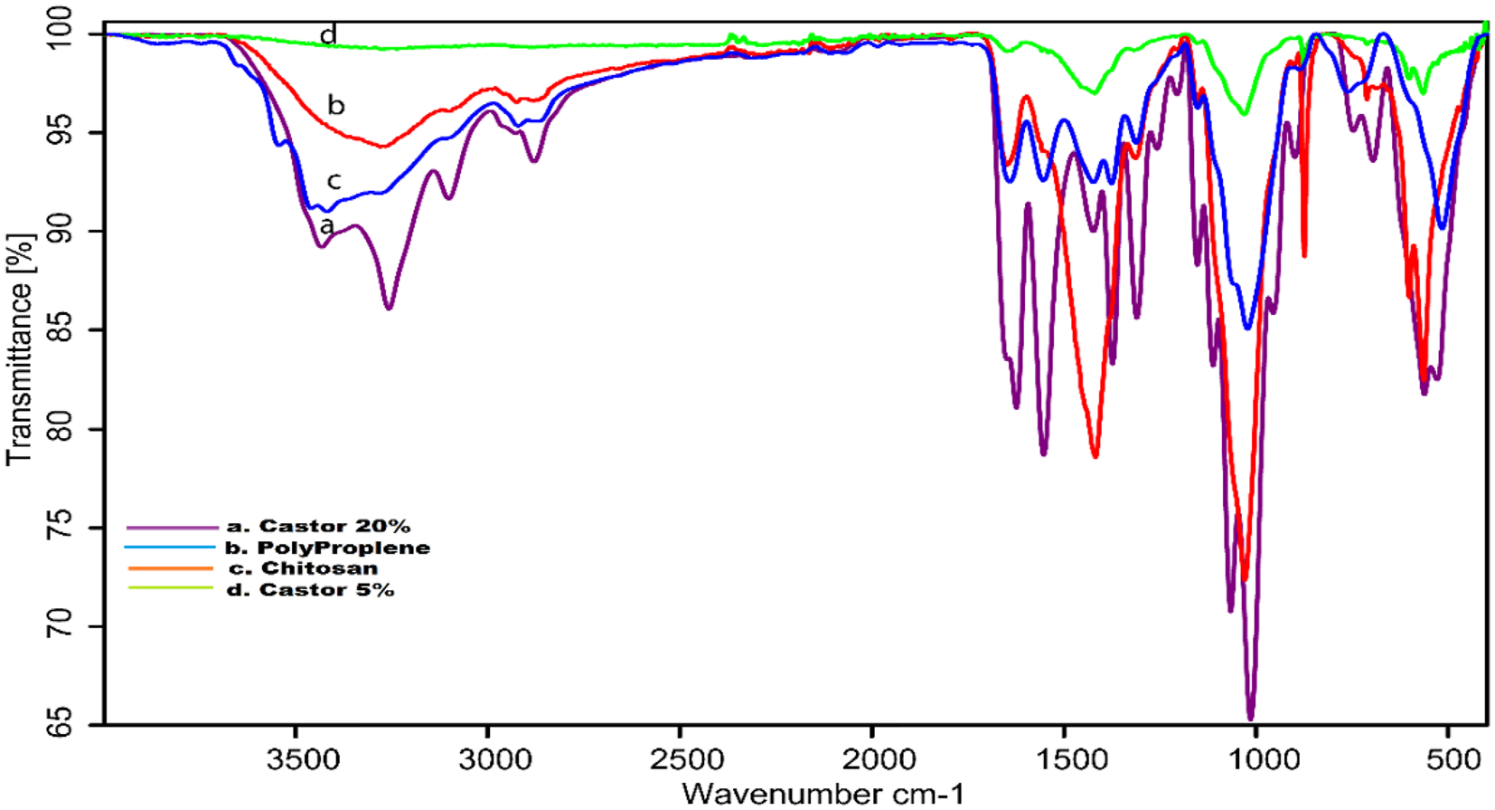
Different FTIR spectra of; chitosan (70%)/starch (10%)/castor (20%) (a), polypropylene (as a petroleum synthetic plastic) (b), extracted chitosan (c), and castor chitosan (90%)/starch (5%)/castor (5%) (d).

**Figure 4:**
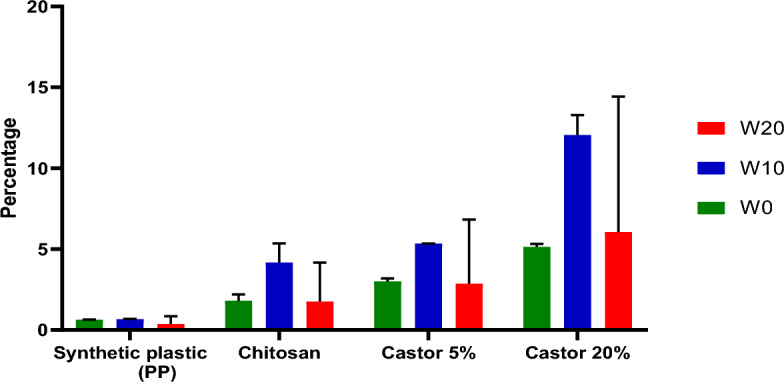
Degree of swelling (water effect) of different bioplastic films synthesized.

The FTIR analysis was used to assess the functional groups present in both extracted and standard chitosan. In Fig. 1A (See them separately in Supplementary data file Figs. 1S–4S), the IR of extracted chitosan can be observed where a strong band in the region 2920.5, 2845.3 and 1463 cm^−1^ corresponds to OH, NH and NH_2_ stretching chemical bond, as well as the intramolecular hydrogen bonds. The weak bands presented 2660, 2663, 2509, 2016, and 1425 cm^−1^ due to C–H, H–C–H and C=O stretching were also detected. According to Kumari et al.^37^, the methyl group in NHCOCH_3_, the methylene group in CH_2_OH, and the methane group in the pyranose ring all exhibit stretching vibrations in the range of 2921–2879 cm^−1^. This was nearly range with our study peaks at 2920.5 and 2845.3 cm^−1^. The absorption bands at around 1076, 719.8 cm^−1^ were medium peaks N–H, C–O, and C–O–C groups, while the characteristic peaks assignment of the extracted chitosan at 3602 and 1799 cm^−1^ was attributed to the stretching vibration of OH chemical bond, also the peak was showed at 492.6 cm^−1^ may be due to Si–O–Si asymmetrical and symmetrical stretching. On the other hand, the FTIR spectrum analysis of standard chitosan was a broad strongest band at 3379 and 1959 cm^−1^ corresponding to the stretching of OH groups in Fig. 1B ^59–62^.

The peak at 2880 cm^−1^ is related to N–H in NH_2_ association in primary amines due to different stretching vibration bands. The peak at 1378 cm^–1^ is due to C–C stretching. However, the observation in this spectrum study is identical to what was seen by Muñoz et al.^55^, even though the absorption bands at 1115 cm^–1^ correspond to the vibration of the C-N bond and confirm the joining of the monomers through *α*-glycosidic links.

### Biofilms formation and characterization

As mentioned before, the biofilms were prepared according to the formulation presented in Table 1. The greatest casted biofilms with perfect strength were selected for further analyses. In general, Fig. 2 represents a side of different products from biofilms based on chitosan, which saved the highest harmony and homogeneity for films and showed flexibility. The most acceptable biofilms were the formulations of; Chitosan (90%)/Starch (5%)/Castor (5%) and Chitosan (70%)/Starch (10%)/Castor (20%) in comparison to extracted chitosan film. Besides that, the polypropylene (PP) film was taken into consideration. Indeed, there was no clear reason for such a result. Coupling chitosan with renewable and abundant agro-resources such as starch can be utilized to improve moisture resistance accelerate degradation, and amazingly reduce the manufacturing cost^63^. Generally, the properties of chitosan movies are influenced by a few components such as atomic mass and degree of deacetylation of chitosan, as well as the nearness or nonappearance of plasticizers^64^. However, one of the polymers that has been thoroughly investigated for application as a biodegradable plastic is biofilm starch-chitosan.

### FTIR of synthesized bioplastic films

Figure 3 shows several FTIR spectra of the synthetic bioplastic sheets. The absorption peaks at 3280 cm^–1^ and 2924.3 cm^–1^ in the spectra of the chitosan film alone are caused by the overlap of NH_2_ and OH^65–68^. The N–H band is responsible for the band absorption band at 1650.4 cm^–1^. The presence of the OH group is indicated by the absorption peak at 1417.69 cm^–1^^65^. There are C-N group attributed to the amino group at an absorption peak of 1313.99 cm^–1^^69^. The absorption peaks at 1153.4 and 1029.2 cm^–1^ indicate the existence of the C–O group^65^. Occurring at 2323.2 cm^−1^ and 2111.49 cm^–1^ are ascribed to the O–H stretching mode in chitosan indicating the existence of the acid group. The absorption band at 1793.79 cm^−1^ is due to the C=O group^65^.

Additionally, data shows the FTIR spectrum of chitosan (70%)/starch (10%)/castor (20%), which was characterized by the presence of amide I, amide II, and amide III bands near 1640.98, 1586.08, and 1421.6 cm^–1^, respectively. The presence of amide can be confirmed by the N–H stretching band near 3357.46 cm^–1^. On the other hand, carbohydrates can be determined by the presence of broad C–O bands near 1148.83 and 1071.05 cm^–1^. There are O–H stretching groups in the region of 2916.54 cm^–1^ indicating the existence of the acid group. Also, there are C–N group attributed to the amino group at an absorption peak of 1375.24 cm^–1^^69^. The peak was shown at 655.7, 562.3 and 515.5 cm^–1^ may be due to Si–O–Si asymmetrical and symmetrical stretching^70–73^.

Moreover, the data explains the FTIR spectrum of chitosan (90%)/starch (5%)/castor (5%), which was characterized by the presence of amide I, amide II, and amide III bands near 1623.92, 1552.91, and 1423.85 cm^–1^, respectively. In addition, the N–H stretching band between 3432.36 and 3257.06 cm^–1^ confirms the presence of amide. On the other hand, the presence of large C–O bands between 1066.04 and 1014.47 cm^–1^, as well as the O–H group at 3257.06 cm^–1^, can be used to identify carbohydrates^65^. It is seen that the O–H group at 3101.30 cm^–1^ broadens. There are O–H stretching groups in the region of 2879.12 cm^–1^ indicating the existence of the acid group. The FTIR spectrum shows a broad change in the region of 3257.06 cm^–1^. This is due to the Maillard reaction within the castor oil where the ester bond underwent a reaction with the carbohydrate^74^. Also, there are C–N groups attributed to the amino group at absorption peaks of 1373.57, 1310.55, 1257.88, 1205.97, 1152.85, and 111.86 cm^–1^^69^. The absorption bands at around 955.70, 899.39 and 746.33 cm^–1^ were medium peaks N–H, C–O, and C–O–C groups. The peak was shown at 695.38, 561.91 and 528.33 cm^–1^ may be due to Si–O–Si asymmetrical and symmetrical stretching. The peaks on 1623 and 1374 cm^–1^ are included in the unique spectrum of PP featuring CH_2_ deformation and symmetric CH_3_ deformation, while the peaks on 3429 and 3256 cm^–1^ are CH stretch spectra. The spectra on 559 and 527 cm^–1^, on the other hand, indicate an isotactic polypropylene band. Finally, the peaks 1555 and 1412.42 cm^–1^ spectrum suggest C–C bending, which is the backbone of PP.

### Swelling capacity of produced biofilm films

Chitosan is hydrophobic, and the swelling behaviors of unplasticized films formed from extracted chitosans are soluble in glacial acetic acid 99%. However, Fig. 4 represents the films of extracted chitosan, chitosan (90%)/starch (5%)/castor (5%), chitosan (70%)/starch (10%)/castor (20%) and PP from 0 to 20 min in the water. Different synthesized biofilms exhibited a high degree of swelling ranging from 0.0 to 12%. It was observed that the degree of swelling of the chitosan (70%)/starch (10%)/castor (20%) had the highest percentage presented at 20 min and reached 12%, while the lowest percentage of the degree of swelling was in the PP had the same time stable consequently. At the same time, the degree of swelling of the chitosan (90%)/starch (5%)/castor (5%) was 2.5% after 20 min, while the absorption capacity of the extracted chitosan film was less than chitosan (90%)/starch (5%)/castor (5%) approximately 1% after 20 min.

According to Tan et al.^75^, the biofilm's resistance property decreases with increasing chitosan content, which suggests a reduced ability of the biofilm to hold onto water. It results from a polymer chain in chitosan that has many –NH_2_ or hydrophobic amine groups, indicating the significant hydrophobicity of the polymer that makes up chitosan. A biopolymer called chitosan might have excellent qualities that make water safe for biofilm. It is brought on by the hydrophobicity (hatred of water) of starch-chitosan properties. Less water is retained in biodegradable plastic the more chitosan is used. It is exhibited at the highest level of water resistance, or at a 40% chitosan addition with the highest level of water assimilation of 0.38%^76^.

### Tensile strength (TS)

Because of this, chitosan-derived materials have poor mechanical qualities, which makes it difficult for them to be used in more difficult applications. Expanding the use of plasticizers is one strategy used to solve this issue^77^. The maximal pull before breaking or ripping is used to determine the biofilm's tensile strength. The goal of this measurement is to ascertain the amount of force needed to draw the film to its fullest extent across all of its surface areas. The results of the TS test for different biofilms are collected in Table 2. There are several significant criteria used to evaluate the mechanical properties of biofilms made from chitosan extracted from different microbial cell walls and/or crustaceans’ shells. Specifically, the different analyses can be summarized in TS mechanical properties of biofilm can be known from the reaction of ductile quality and elongation tests. Tensile Strength is the greatest constraint that might be stood up to by a biofilm until breakage^78^. The TS value increased in proportion to the increasing chitosan concentration. This is because more hydrogen bonds form in the biofilm at higher chitosan concentrations, making them stronger and more difficult to break. After all, more energy is required to do so^79^. Based on the data in Table 2, the TS of biofilm samples is influenced by the variation of the composition of chitosan, where in extracted chitosan film high tensile strength was found at 5.71% MPa. Based on the TS test findings, it was determined that adding starch and castor oil to chitosan films significantly increased the value of the TS biofilm.

**Table 2:** Tensile strength, elasticity, young modulus, and stress at the breaking of synthesized bioplastic films were acceptable.

Tested biofilm	Tensile strength (Mpa)	Elongation (%)	Young’s modulus	Stress breaking	Film thickness
Extracted chitosan	5.71 ± 0.05	10.40 ± 0.11	0.002	0.0019	1.22 mm
Chitosan (90%)/starch (5%)/Castor (5%)	5.88 ± 0.08	9.00 ± 0.09	0.050	0.0580	1.21 mm
Chitosan (70%)/starch (10%)/castor (20%)	5.51 ± 0.03	5.92 ± 0.08	0.005	0.0017	1.23 mm
Polypropylene	1.70 ± 0.04	121.00 ± 2.11	0.009	0.0095	1.19 mm

**Figure 5:**
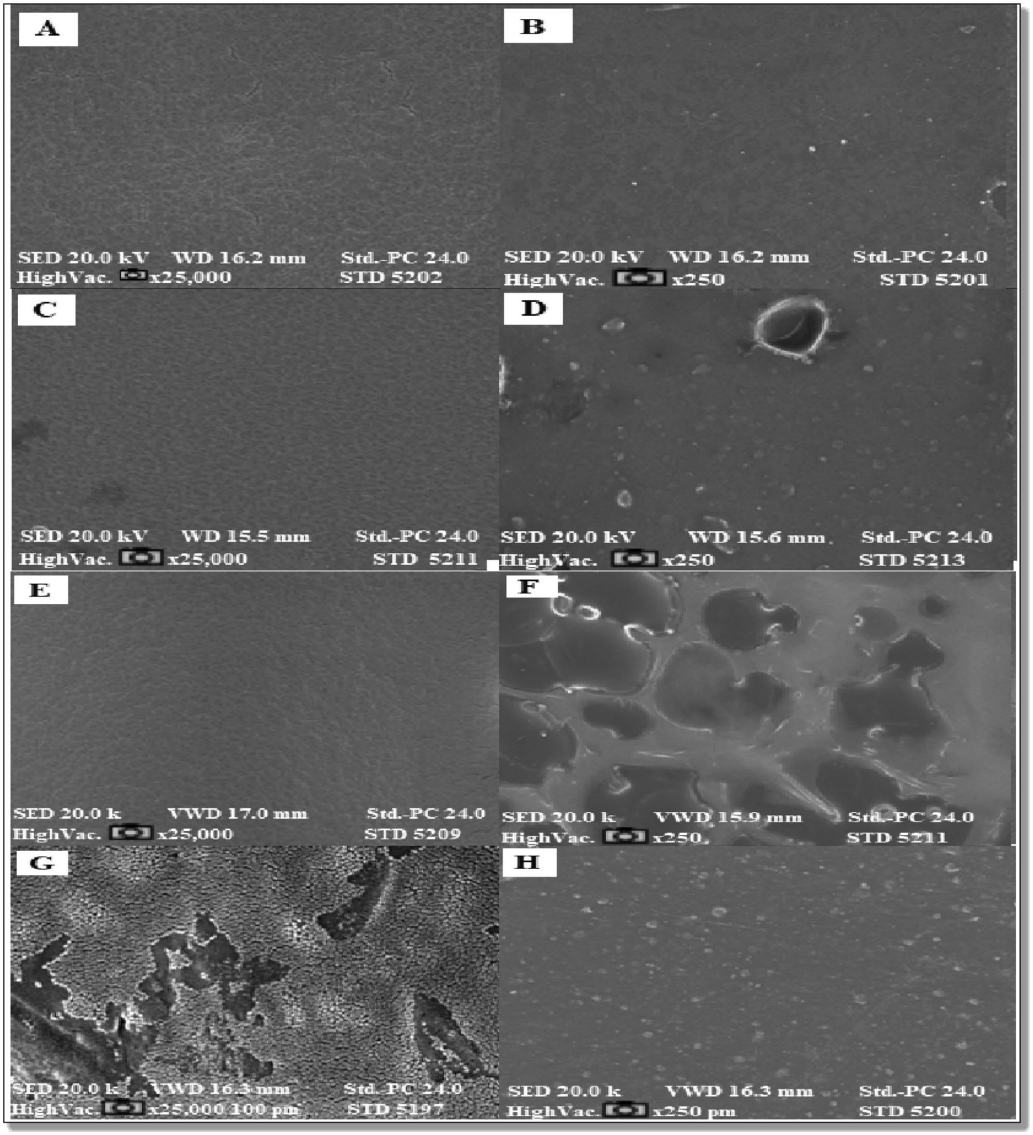
Scanning electron microscope showed (**A**, **B**) extracted chitosan film with different magnifications 25 × 10^3^ and 250, respectively, (**C**, **D**) presented castor oil 5% mixed with starch-chitosan complex with 25 × 10^3^, 250 dramatically, (**E**, **F**) also castor oil 20% mixed with starch-chitosan complex as 25 × 10^3^ and 250 magnifications, finally (**G**, **H**) given as standard polypropylene bag at 25 × 10^3^ and 250 consequently.

The elongation at the break of the isolated chitosan film was 10.4%. The percentage change in the specimen's initial length between the grips of a film to stretch (extend) reveals the flexibility and elongation capacity of the film, which is calculated at the point at which the film breaks during tensile testing. Young's modulus measures how rigid the film is, and a lower value denotes a more flexible substance. Chitosan was added, and this resulted in a considerable decrease in the young's modulus and the development of more flexible films. In the addition of chitosan, the Young's modulus, which was 0.002 MPa, decreased. In addition, the elongation for the chitosan (90%)/starch (5%)/castor (5%) was breaking at 9.0%, and that shows the film capacity for flexibility, the rigidity of the film determined by Young’s modulus was 0.05 MPa. As well as the biofilm made of chitosan (70%)/starch (10%)/castor (20%) appears to have the maximum tensile strength at 5.51 MPa. The films' ability to elongate and bend may be seen by looking at the elongation at break, which was 5.92%. The inclusion of chitosan decreased the rigidity of the film, as measured by Young's modulus, which was 0.005 MPa and the breaking point was 0.0017 Mpa. Further, the data illustrates the TSA stress–strain relationship of PP. The behavior of all curves is essentially the same; they all start linearly, defining the slope, and then they all start to taper off after a given amount of strain, showing the stiffness of the material as it gets closer to the failure load. These plots are used to extract all significant tensile parameters, which are then provided in terms of the average, upper and lower bounds for the tensile modulus, strength, and maximum elongation. This resulted in a higher diameter of elongation which was 10.8%, lower Young’s modulus and TSA, and higher residual strain as well at 0.009 Mpa, and the breaking point was 0.0095 Mpa. The elongation of polypropylene decreases with the rise in the natural filler loading in the natural rubber composites, the PP was breaking at 11.2% (See them separately in Supplementary data file Fig. 5S, 6S, 7S, 8S).

The addition of up to 30% chitosan results in the maximum value of TS, which is 38.25 MPa. The important aspect that affects the mechanical behavior of biofilm material, according to Fathanah et al.^80^, is an affinity between its constituent parts. A certain atom or molecule may have a propensity to form bonds due to a phenomenon called affinity. The more molecules that bind, the stronger the affinity. The chemical bonding of a biofilm's constituents has an impact on its strength. The number of molecules that are bound and the type of binding determine how strong a chemical is. It takes a lot of energy to break a strong chemical bond because strong chemical bonds are difficult to break.

### Thermogravimetric analysis (TGA)

The curve of the extracted chitosan shows two stages of weight loss between 50 and 400 °C, with the first stage occurring between 50 and 100 °C due to water molecule loss and resulting in a weight loss of roughly 10.9%. A weight loss of about 41% resulted from the primary deterioration of the extracted chitosan, which started at 220 °C and was destroyed at 400 °C. The TGA of the chitosan (90%) / starch (5%) / castor (5%) showed three different stages of weight loss. The loss of adsorbed water may correspond to the initial stage of weight loss of 10.38%, which occurs at 60 °C, the thermal degradation causes the second disintegration step, which results in a weight loss of roughly 25.52% in the range of 250 °C. The third degradation was at approximately 300–400 °C because of thermal degradation with weight loss of about 25.8%. The TGA curve of chitosan (70%)/starch (10%)/castor (20%) exhibited that three stages 190 to 450 °C. The first stage results in a weight loss of 5.94% at 190 °C. The second decomposition stage occurs at 230–330 °C due to thermal degradation with a weight loss of about 16.6%. The third degradation was at approximately 350–500 °C because of thermal degradation with weight loss of about 56.1%. Furthermore, the TGA analysis for the PP film sample contains two stages in mass: the first stage indicates the thermal degradation of 3.09% occurs at 450 °C and the second stage is the thermal decomposition at 84.6% at 450 °C (See them separately in Supplementary data file Figs. 9S–12S).

### TGA–DSC study

The DSC of extracted chitosan shows two broad endothermic peaks at 90.7 °C and the exothermic decomposition peaks at onset at 282.3–547.8 °C. The DSC of chitosan (90%)/starch (5%)/castor (5%) exhibited the broad endothermic peaks attributable to water loss at approximately 92.2 °C, the exothermic decomposition peaks at onset at 232.9–650 °C. As well as, the DSC thermogram of the films collected during the first heating cycle showed three signals endothermic peaks below 100 °C at 98.4 °C, while the exothermic peaks are broad variations 293, 379.6 and 645.5 °C. In addition, The TGA of the chitosan starch with castor 5% showed three different stages of weight loss. The loss of adsorbed water may correspond to the initial stage of weight loss of 10.38%, which occurs at 60 °C, the thermal degradation causes the second disintegration step, which results in a weight loss of roughly 25.52% in the range of 250 °C. The third degradation was at approximately 300–400 °C because of thermal degradation with weight loss of about 25.8%. Moreover, the TGA result of chitosan (70%)/starch (10%)/castor (20%) exhibited that shows that three stages 190 to 450 °C. The first stage results in a weight loss of 5.94% at 190 °C. The second decomposition stage occurs at 230–330 °C due to thermal degradation with a weight loss of about 16.6%. The third degradation was at approximately 350–500 °C because of thermal degradation with weight loss of about 56.1%. Furthermore, the TGA analysis for the PP sample contains two stages in mass: The first stage indicates the thermal degradation in 3.09% occurs at 450 °C and the second stage is the thermal decomposition at 84.6% at 450 °C (See them separately in Supplementary data file Figs. 13S–16S).

### X-ray diffraction (XRD)

To design a particular application in the field of packaging industries crystallinity is one of the important 'criteria for food packaging films. The X-ray diffraction measurements were carried out to analyze the nature of each film sample of extracted chitosan film, chitosan (90%)/starch (5%)/castor (5%), chitosan (70%)/starch (10%)/castor (20%) and PP as shown in Figs. 17S–20S, respectively. The X-ray patterns of chitosan film blends exhibited diffraction peaks of chitosan at around 9°, 20° and 64° of 2*θ*. When compared with Tripathi et al., X-ray patterns of chitosan. The diffraction peak of chitosan is at around 10° and 20° of 2θ. the highest intensity of about 800 counts at 2θ = 19°^81^. Moreover, the X-ray diffraction patterns of chitosan (90%)/starch (5%)/castor (5%) showed diffractive peaks in the region from 9°, 20° and 64°of 2θ with the highest intensity about 280 counts at 2θ = 20° than chitosan 220 counts. Furthermore, the XRD diffraction studies chitosan (70%)/starch (10%)/castor (20%), there are four peaks presented around 2θ = 9°, 2θ = 28° and sharp peaks at 2θ = 20°, 2θ = 43.5°. The highest intensity about 440 counts around 43.5° of 2θ. While the XRD patterns of PP were broad peaks at 2θ = 20°, 2θ = 23° and small peaks at 2θ = 9°, 2θ = 29°, 2θ = 36°, 2θ = 43.5°, and 2θ = 64°. Also, the highest intensity is about 1350 counts in 2θ = 23° (See them separately in Supplementary data file Figs. 17S–20S).

### Surface area analysis

The Adsorption–desorption isotherm of Chitosan (70%)/Starch (10%)/Castor (20%)was studied as an example and the data were reported in the supplementary data (Figs. 21S–23S). The BET and BJH analysis showed that the film is mesopore with 3.1 nm mean pore diameter and 11.74 m^2^/g specific surface area.

### Morphological analysis

In only a few straps, the chitosan film was uniform and continuous, with a high basic and no interface layer, as illustrated in various SEM micrographs of removed chitosan surfaces and manufactured bioplastic films in Fig. 5. This displays both the consistency and compatibility with which chitosan chains are depicted throughout the films. In general, hydrogen bonding between the functional bunches of the mixed component (–Gracious and –NH_2_ bunches in chitosan) was what caused the homogeneous mixtures of extracted chitosan-starch and castor oils, in both percentages; 5 and 20%. The SEM images at a magnification of 25 × 10^3^ were obtained. When viewed on a computer, it was clear that they had accurately shown every pore on the pore surface and that, in addition to the PP scaffold membrane surface, the extracted chitosan and bioplastic films also had unevenly distributed pores. By SEM to watch the morphology of the break surface of the biofilms, and whether all components of biofilms have been blended homogeneously. A nonporous, smooth membranous phase with dome-shaped orifices, crystallite, and microfibrils was visible in the SEM images of the extracted chitosan film. Both chitosan (90%)/starch (5%)/castor (5%) and chitosan (70%)/starch (10%)/castor (20%) derivative electron micrographs showed that they had a porous, chain-like structure. Further, these images demonstrated the close spherical morphology of our synthesized biofilms, which may be utilized in biomedical applications^37^.

### Antimicrobial activity of bioplastic films

There are several methods to evaluate the antimicrobial activity of the biofilms. This research used a disc diffusion test as an antimicrobial activity test for our bioplastic film but no clear result was recorded. It may be due to the weak contact between the bioplastic films and reference strain in the agar medium. Therefore, the antimicrobial activity was re-evaluated via suppression percentages of reference strains in liquid broth as mentioned in the “Materials and methods” section.

In particular, the data presented in Table 3 confirmed that there was no suppression detected after 2 h against any reference strain used (*E. feacalis*, *K. pnumina*, *B. subtilis*, and *P. aeruginosa*). It was also observed that the positive values of suppression % ranged from 14.8 to 96.8%. The lowest suppression was recorded for biofilm of chitosan (70%)/starch (10%)/castor (20%) against *K. pnumina*, while the highest one was recorded for the same formula against *B. subtilis*. From this result, the most susceptible reference strain was *B. subtilis*, while the most resistant reference strain was *K. pnumina*. Specifically, our bioplastic films have possessed effective antimicrobial activities. Thus, they are promising in such a manner with additional value.

**Table 3:** Suppression percentages (%) of different effective formulas of extracted chitosan and synthesized bioplastic films detected after four incubation intervals (2, 4, 12, and 24 h).

Incubation intervals (h)	Bioplastic film	Suppression percentage (%)			
		*E. feacalis*	*K. pnumina*	*B. subtilis*	*P. aeruginosa*
After 2 h	Extracted chitosan	No suppression	No suppression	No suppression	No suppression
	Castor 5%	No suppression	No suppression	No suppression	No suppression
	Castor 20%	No suppression	No suppression	No suppression	No suppression
After 4 h	Extracted chitosan	No suppression	14.8 ± 1.11	No suppression	No suppression
	Castor 5%	No suppression	26.7 ± 1.92	No suppression	No suppression
	Castor 20%	No suppression	14.7 ± 1.03	No suppression	No suppression
After 12 h	Extracted chitosan	39.6 ± 1.12	88.0 ± 2.42	86.6 ± 3.11	62.4 ± 0.92
	Castor 5%	82.1 ± 2.52	77.0 ± 1.81	87.2 ± 2.15	59.2 ± 0.86
	Castor 20%	82.4 ± 3.11	79.4 ± 1.91	89.4 ± 2.41	70.0 ± 1.18
After 24 h	Extracted chitosan	96.1 ± 3.65	84.8 ± 2.13	94.7 ± 1.57	80.0 ± 1.24
	Castor 5%	94.7 ± 3.18	80.4 ± 2.51	93.6 ± 1.69	77.9 ± 1.31
	Castor 20%	92.9 ± 3.22	81.4 ± 2.63	96.8 ± 2.35	79.2 ± 1.17

Initially, chitosan has good properties to be moulded into plastic and has antimicrobial properties because it is easily biodegraded and biocompatible when combined with other materials and possesses biofunctional characteristics^18^. Consequently, chitosan has been utilized as a material for food packaging to preserve food quality^82^.

The development of chitosan-based solutions was the subject of a lot of research since the inherent qualities of chitosan might improve the packaging's antimicrobial effectiveness. Due to chitosan's ability to form films, several natural polysaccharides have been combined to create film packaging materials. Cha and Chinnan^20^ illustrated when they examined the antibacterial action of a chitosan-polysaccharides film against Listeria monocytogenes and showed that the growth of the bacteria was completely inhibited. The antibacterial activity of chitosan-HPMC films was significantly reduced when the amino groups of chitosan were cross-linked with citric acid, revealing the vital importance of the protonated amino groups of chitosan for antimicrobial action. Moreover, the biofilms prepared by Agustin and Padmawijaya^83^ showed fair antibacterial activity against *S. aureus* and *E. coli*. Recently, Chang et al.^84^ prepared chitosan-based plastic films, which had high antibacterial activity; especially with an inhibitory efficiency of over 95% against *E. coli*, *P. fluorescens*, and *S. aureus*.

Numerous studies have demonstrated that additional -NH^3+^ residues in chitosan make it easier for it to bind to bacteria, which causes structural instability in the bacteria^85^. However, as the amount of chitosan in the film rises, it may tighten the film's structure, prevent amine groups from being exposed, or create electrostatic attraction between or between chitosan molecules, decreasing the antibacterial activity of the material^84^. Nevertheless, the addition of a plasticizer into chitosan biofilms may decrease the antimicrobial activity of biofilms because it fills the space between hydrogen bonds and will weaken the chitosan strength which can affect chitosan antimicrobial activity.

### Biodegradation of bioplastic films

The biodegradability of the generated biofilm was evaluated to gauge its eco-friendliness. The outcomes shown in Table 4 supported the biodegradability of the synthetic biofilms we created, with weight loss during burial indicating genuine degradation. However, after the experiment, bioplastic films made with isolated chitosan became lighter and more translucent. In the case of the chitosan (90%)/starch (5%)/castor oil (5%) recipe, the biofilm's color faded and it also became lighter and pore-filled. Various biofilm formulations become clear and cutting-friendly. Observably, PP as a synthetic plastic was used to compare all previous bioplastic films, and this has not changed at all. All of the examined bioplastic films lost weight over time, except PP, which essentially maintained its weight in all treatment media. On day 7, it was discovered that both films had lost more than 40% of their original weight. Chitosan-reinforced starch-based biofilm decomposed by 53.6%, while pure starch-based biofilm film deteriorated by 47.5%. No appreciable weight loss was observed for any biofilm the second and third weeks saw a 47.5% degradation of the biofilm^75^. As well as, day 28 marked the end of the weight loss of pure starch-based and chitosan-reinforced starch-based and 52.1 and 67.7%, respectively, of the total. Weight reduction was greater with the starch-only biofilm than with the chitosan-reinforced version.

**Table 4:** Biodegradability of the formed bioplastic films (2 × 2 cm^2^) from different formulations along two weeks of incubation in different environments.

Formula of biofilm films	Evaluation/parameter	Treatment/weight of biofilm (mg)				
		Seawater (SW)	Marine sediment (MS)	Municipal wastewater (MW)	Garden soil (GS)	Municipal house waste (HW)
Chitosan pure 100% (extracted in this study)	At zero-day	9.1 ± 0.05	7.5 ± 0.07	10.3 ± 0.01	12.9 ± 0.03	8.3 ± 0.05
	After 4 days	8.9 ± 0.12	6.3 ± 0.09	9.2 ± 0.12	9.5 ± 0.06	8.1 ± 0.07
	After 7 days	3.1 ± 0.07	3.5 ± 0.13	3.8 ± 0.2	3.7 ± 0.08	3.2 ± 0.23
	After 14 days	2.8 ± 0.15	2.2 ± 0.2	1.8 ± 0.09	1.7 ± 0.11	1.1 ± 0.19
Chitosan starch added 5% castor	At zero-day	4.1 ± 0.01	3.2 ± 0.19	3.5 ± 0.15	3.4 ± 0.25	4. 2 ± 0.11
	After 4 days	4.0 ± 0.21	6.7 ± 0.11	12.3 ± 0.35	2.8 ± 0.5	3.3 ± 0.09
	After 7 days	3.1 ± 0.09	11.0 ± 0.16	7.0 ± 0.05	3.3 ± 0.24	3.2 ± 0.21
	After 14 days	2.3 ± 0.33	8.4 ± 0.11	7.0 ± 0.09	3.1 ± 0.15	1.3 ± 0.05
Chitosan-starch/castor 20%	At zero-day	6.0 ± 0.05	6.3 ± 0.17	6.6 ± 0.23	6.3 ± 0.08	6.8 ± 0.01
	After 4 days	6.2 ± 0.14	10.0 ± 0.16	8.2 ± 0.31	3.7 ± 0.13	3.2 ± 0.11
	After 7 days	3.8 ± 0.19	8.9 ± 0.05	4.8 ± 0.14	3.7 ± 0.17	2.1 ± 0.05
	After 14 days	2.5 ± 0.23	5.7 ± 0.11	2.1 ± 0.2	2.8 ± 0.18	1.3 ± 0.2
Market plastic (polypropylene, PP)	At zero-days	3.3 ± 0.12	3.7 ± 0.01	3.5 ± 0.05	3.0 ± 0.05	3.2 ± 0.15
	After 4 days	3.3 ± 0.09	3.7 ± 0.15	2.1 ± 0.5	2.0 ± 0.09	2.1 ± 0.3
	After 7 days	3.2 ± 0.05	3.5 ± 0.09	2.6 ± 0.23	2.5 ± 0.25	1.7 ± 0.17
	After 14 days	3.0 ± 0.05	3.5 ± 0.2	1.3 ± 0.15	2.9 ± 0.05	1.3 ± 0.09

Starch-based biofilm layers during burial may be related to higher starch matrix hydrophilicity^86^. The biofilm made of starch and chitosan exhibited a slower rate of deterioration, possibly because chitosan has antibacterial characteristics. In addition, the hydrogen bonding interaction between starch and chitosan, may lowered the starch matrix's hydrophilicity, which slowed the biodegradation^87^.

The most important variables in biodegradation are more particularly the physiochemical characteristics of the biodegradable polymers, the ambient circumstances, and the microbial populations to which the biofilms are exposed. This process can take place in both natural and artificial settings, under aerobic and anaerobic circumstances latter of which has received the least attention in the literature. Compost, soil, and a few aquatic habitats are among the researched aerobic environments, whereas anaerobic environments include anaerobic digestion plants and a few aquatic habitats^16^. However, the breakdown process is influenced by both biotic (microbial activity) and abiotic (UV radiation, temperature, moisture, and pH) factors^88^. The thickness of the biodegradable material is another element that affects the rate of biodegradation; the thicker the product, the longer it will take to degrade^89^.

## Conclusion

Chitosan was extracted from the chitin-wastes of *A. antennatus*, which was locally collected. The quality of the extracted chitin and chitosan yield, moisture content, solubility, and ash were determined and found to be of good quality. Starch and castor oil were used to produce three different bioplastic film formulations, each consisting of ten different composites, with chitosan being used only as a positive control. Our biofilms showed high swelling capacity, while synthetic plastic polypropylene exhibited low swelling percentages. The bioplastic films were identified by FTIR profiles and were found to have acceptable characteristics based on DSC, TGA, and XRD results. SEM images showed that the bioplastic films formed were homogeneous and continuous with a few straps of chitosan film, while the homogeneous mixes of chitosan and castor oil with 5 and 20% were caused by the intelligence hydrogen bonds between the utilitarian bunches of the mixed component. Additionally, the bioplastic formulas exhibited antibacterial activity against common bacterial pathogens (*E. faecalis*, *K. pneumoniae*, *B. subtilis*, and *P. aeruginosa*). The bioplastic films also demonstrated effective antimicrobial activity. The biodegradability of the bioplastic films was evaluated in various environments over several weeks and found to have reduced in weight compared to synthetic plastic polypropylene, which did not change at all. However, it slightly increased material durability to degrade. These results show promise for many applications, particularly in the food packaging industry.

### Supplementary Information

Supplementary Figures.

## Data Availability

The datasets used in this investigation are accessible for review upon request from the corresponding author of the paper.
