# Supplementary material for: Biodegradable plastic formulated from chitosan of Aristeus antennatus shells with castor oil as a plasticizer agent and starch as a filling substrate
Source: Sci Rep. 2024 May 15;14:11161. doi: 10.1038/s41598-024-61377-9 (PMC11096362; doi:10.1038/s41598-024-61377-9)
Supplement: Supplementary file 1 — Supplementary Figures. [file 41598_2024_61377_MOESM1_ESM.docx]

**Supplementary data**

**Biodegradable plastic formulated from chitosan of *Aristeus antennatus* shells with castor oil as a plasticizer agent and starch as a filling substrate**

Ayaat R. El Feky^1^, Mohammed Ismaiel^1^, Murat Yılmaz^2^, Fedekar M. Madkour^1^, Ahmed El Nemr^3*^, Hassan A.H. Ibrahim^3^

**
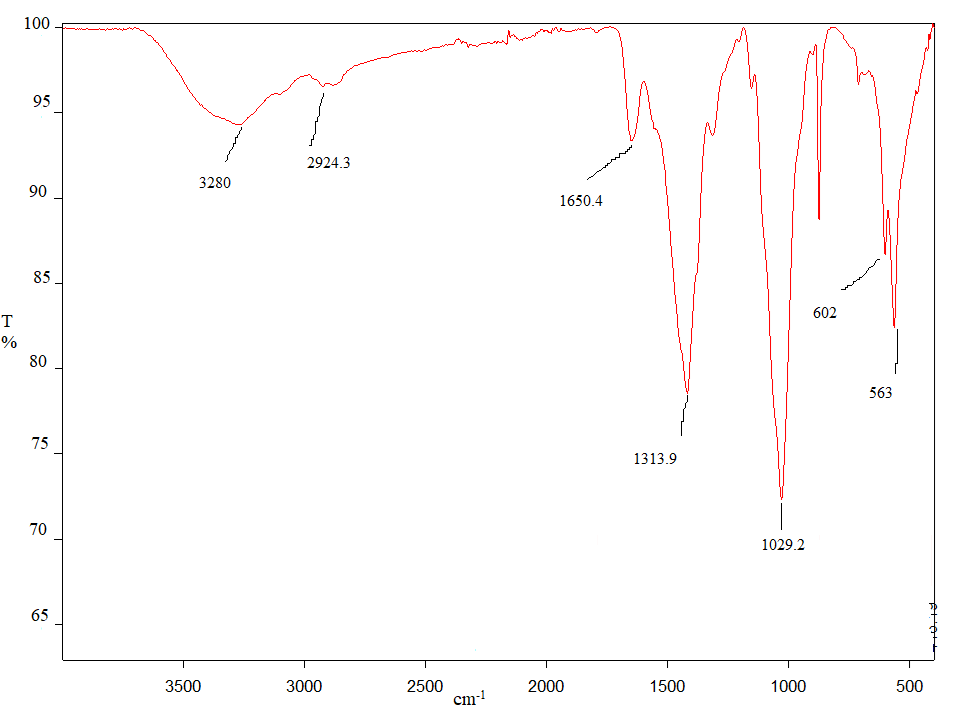
**

**Figure 1S:** FTIR spectrum of extracted chitosan film.


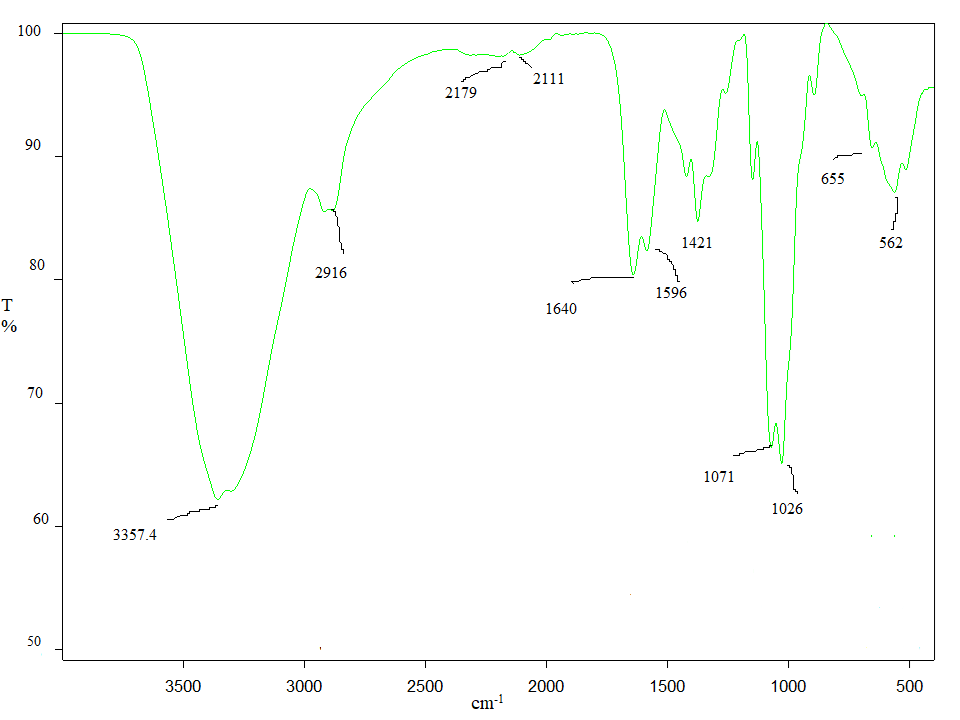


**Figure 2S:** FTIR spectrum of formula; Chitosan (90%) / Starch (50%) / Castor (5%).

#
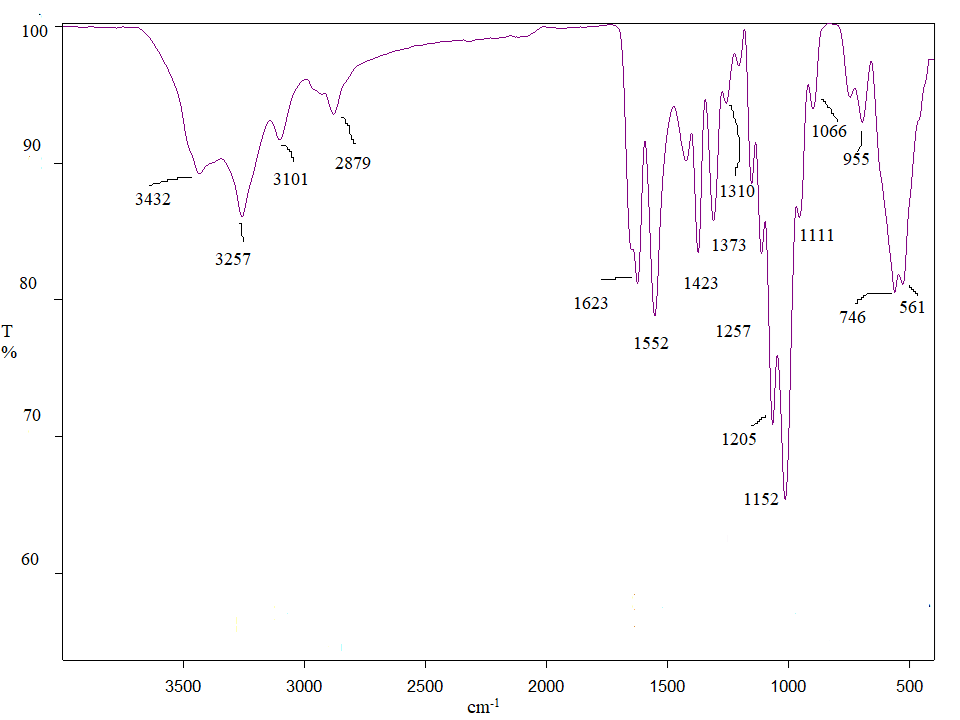


**Figure 3S:** FTIR spectra of formula; Chitosan (70%) / Starch (10%) / Castor (20%).

**
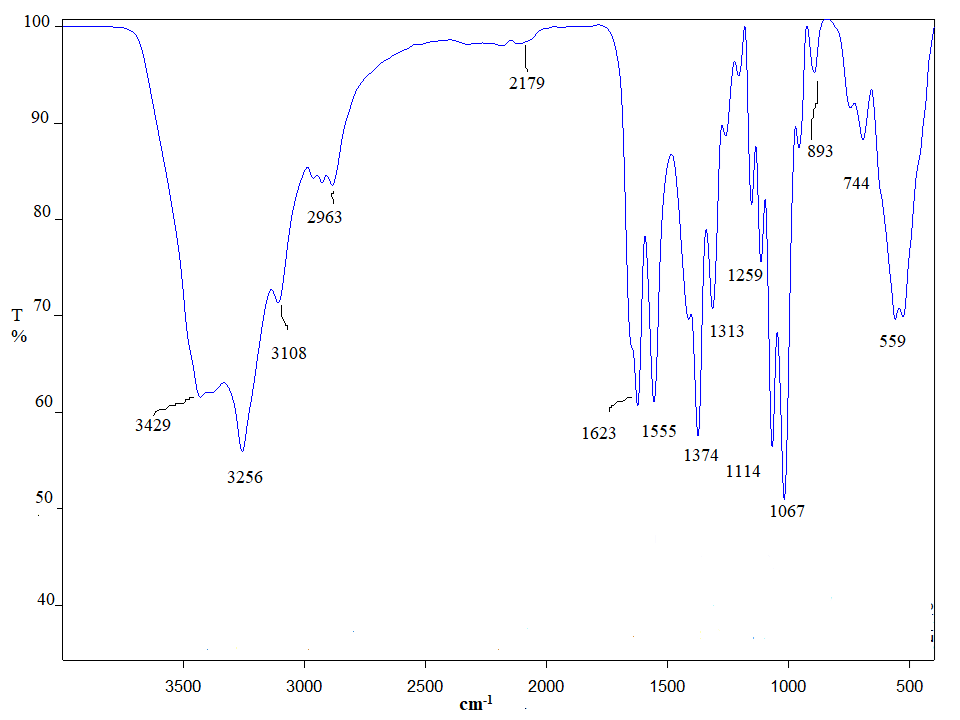
**

**Figure** **4S:** FTIR spectrum of polypropylene (PP) film.


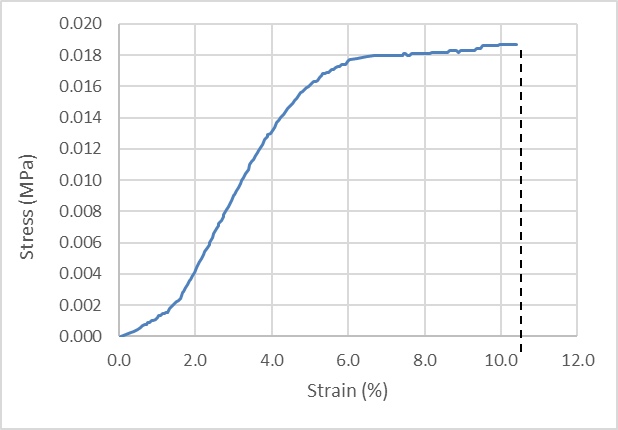

**Figure 5S:** Tensile strength of extracted chitosan film.


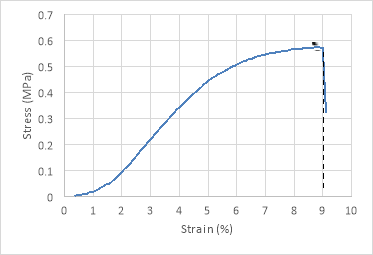

**Figure 6S:** Tensile strength of formula; Chitosan (90%) / Starch (5%) / Castor (5%).


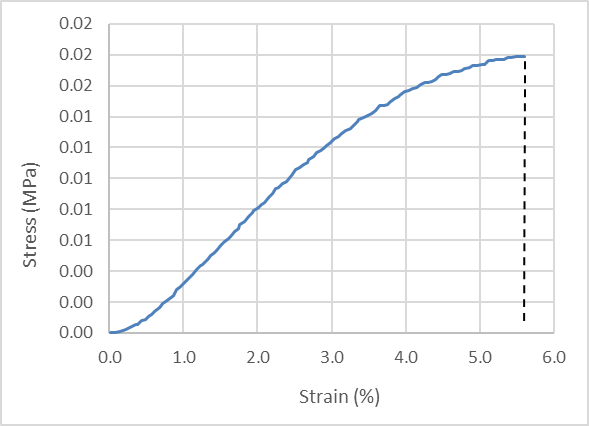


**Figure 7S:** Tensile strength of formula; Chitosan (70%) / Starch (10%) / Castor (20%).

**Figure 8S:** Tensile strength of polypropylene (PP) film.

**
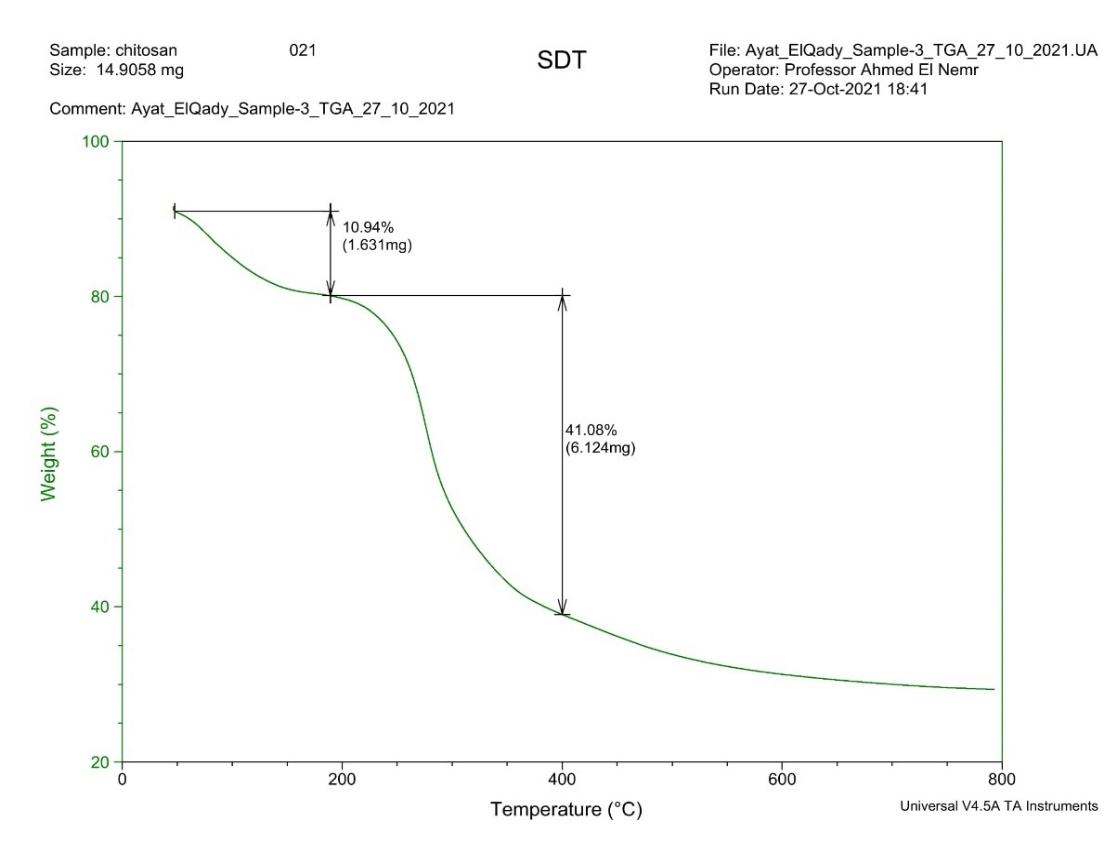
**

**Figure 9S:** TGA of extracted chitosan film.

**
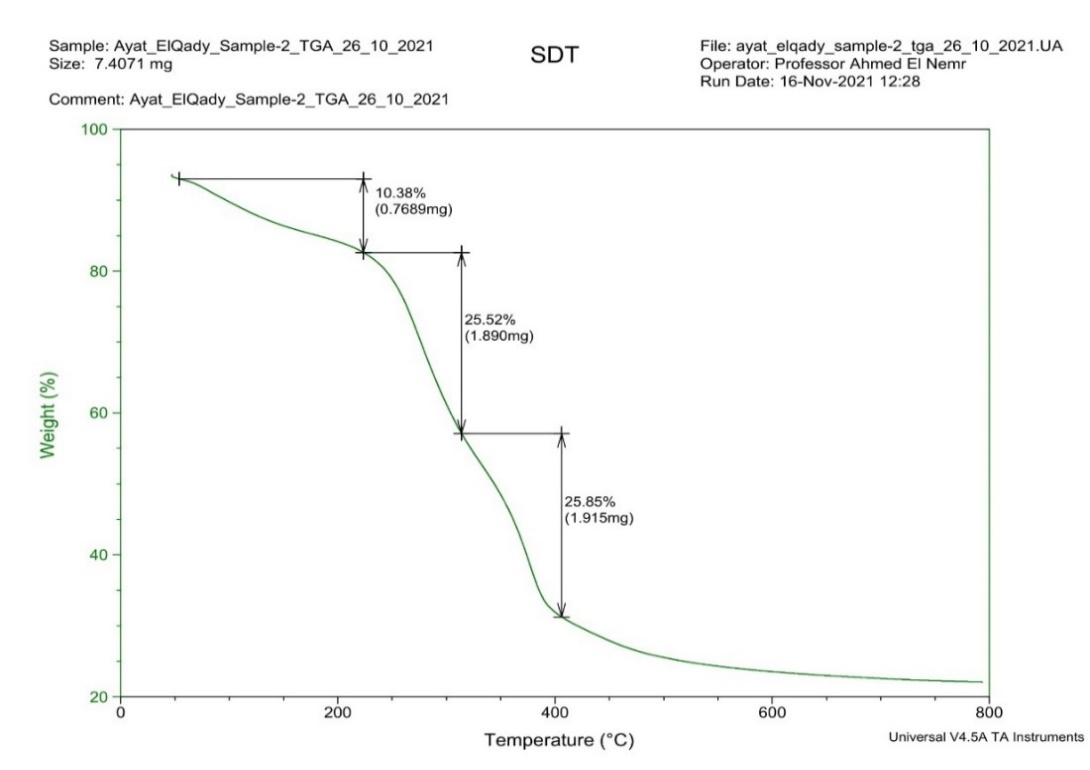
**

**Figure 10S:** TGA of formula; Chitosan (90%) / Starch (5%) / Castor (5%).

**
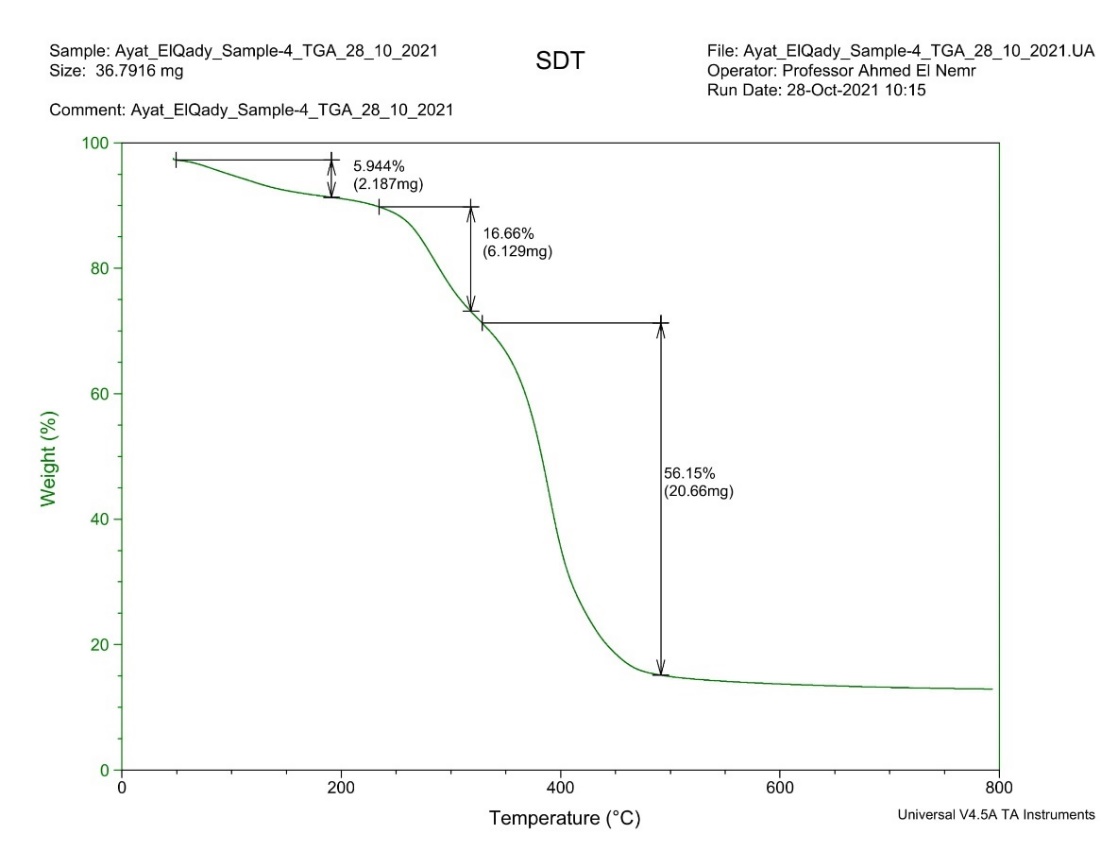
**

**Figure 11S:** TGA of formula; Chitosan (70%) / Starch (10%) / Castor (20%).

**
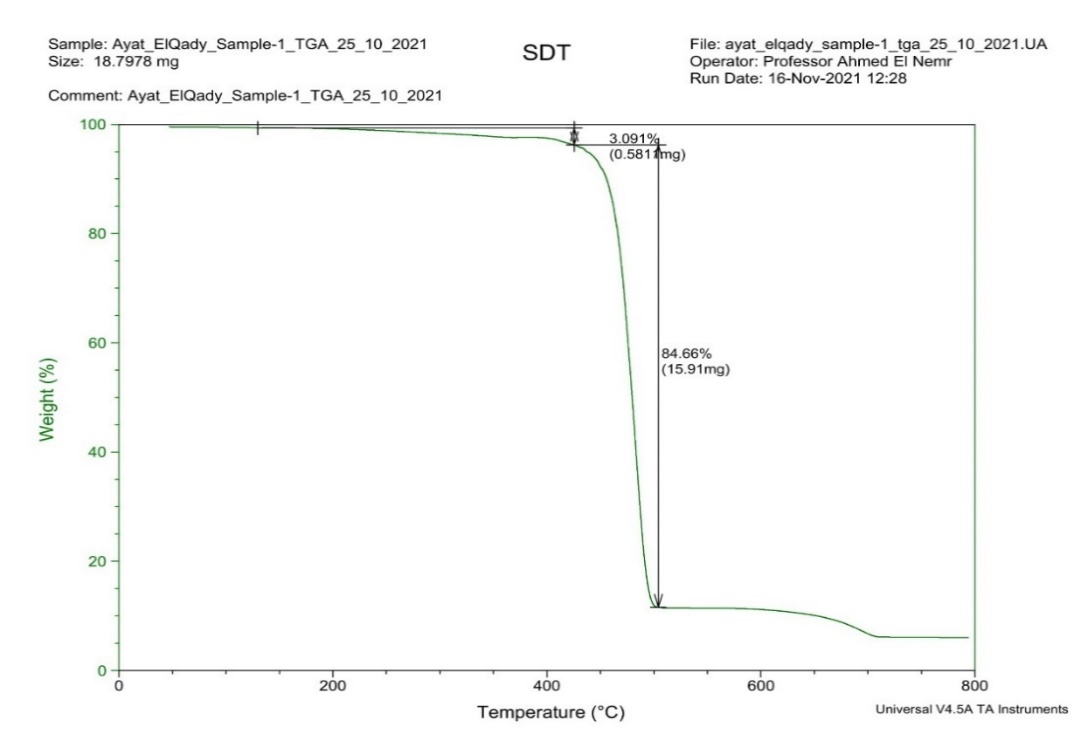
**

**Figure 12S:** TGA of polypropylene (PP) film.

**
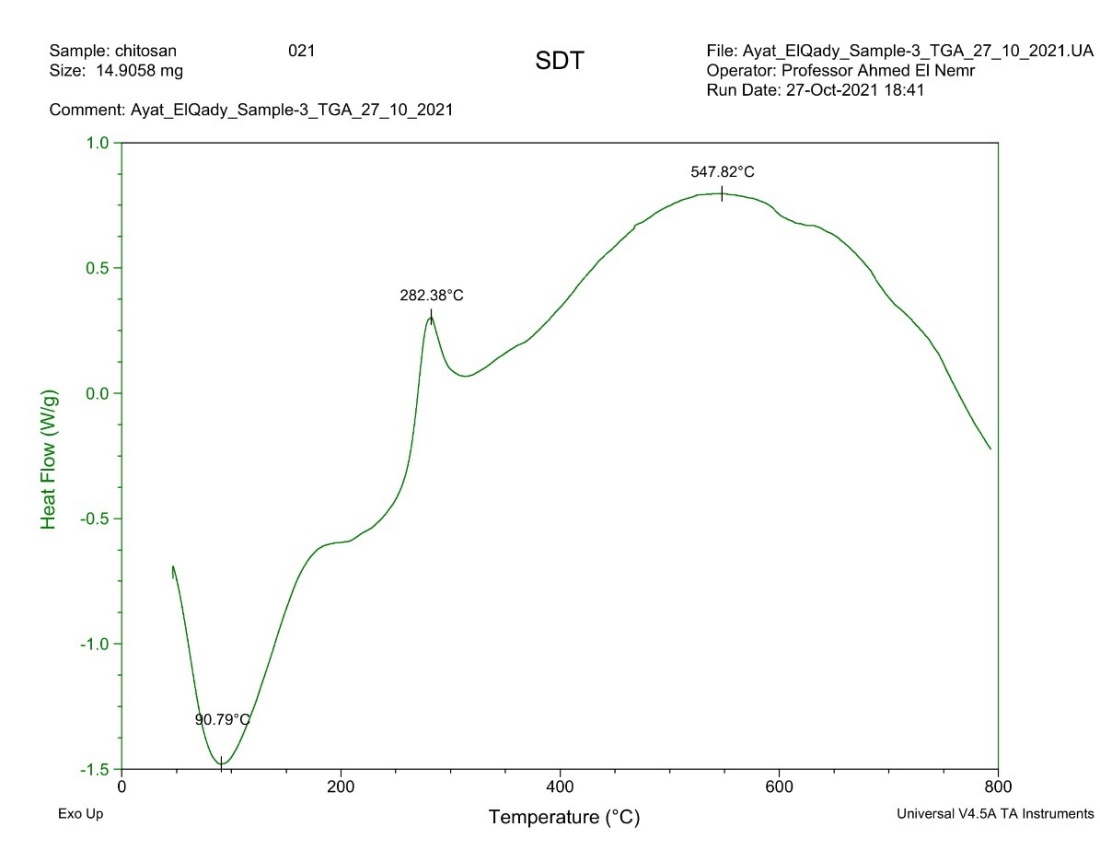
**

**Figure 13S:** DSC of extracted chitosan film.

**
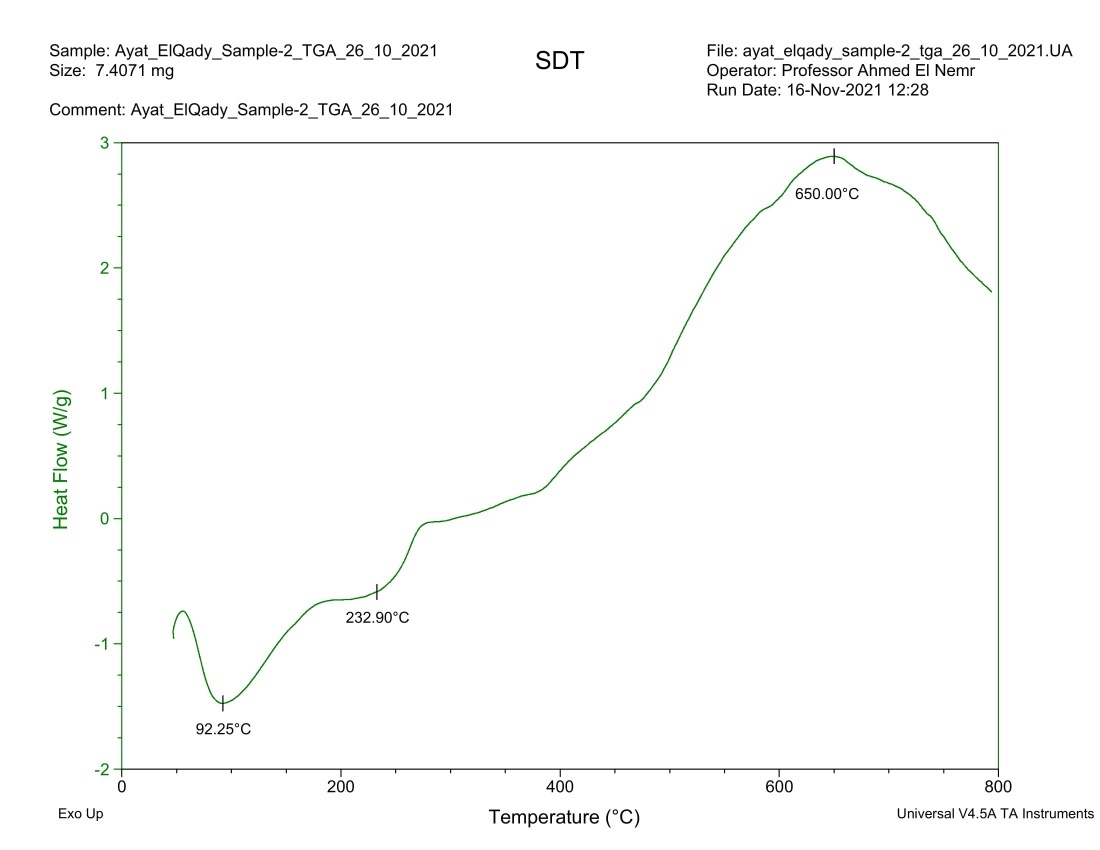
**

**Figure 14S:** DSC of formula; Chitosan (90%) / Starch (5%) / Castor (5%).

**
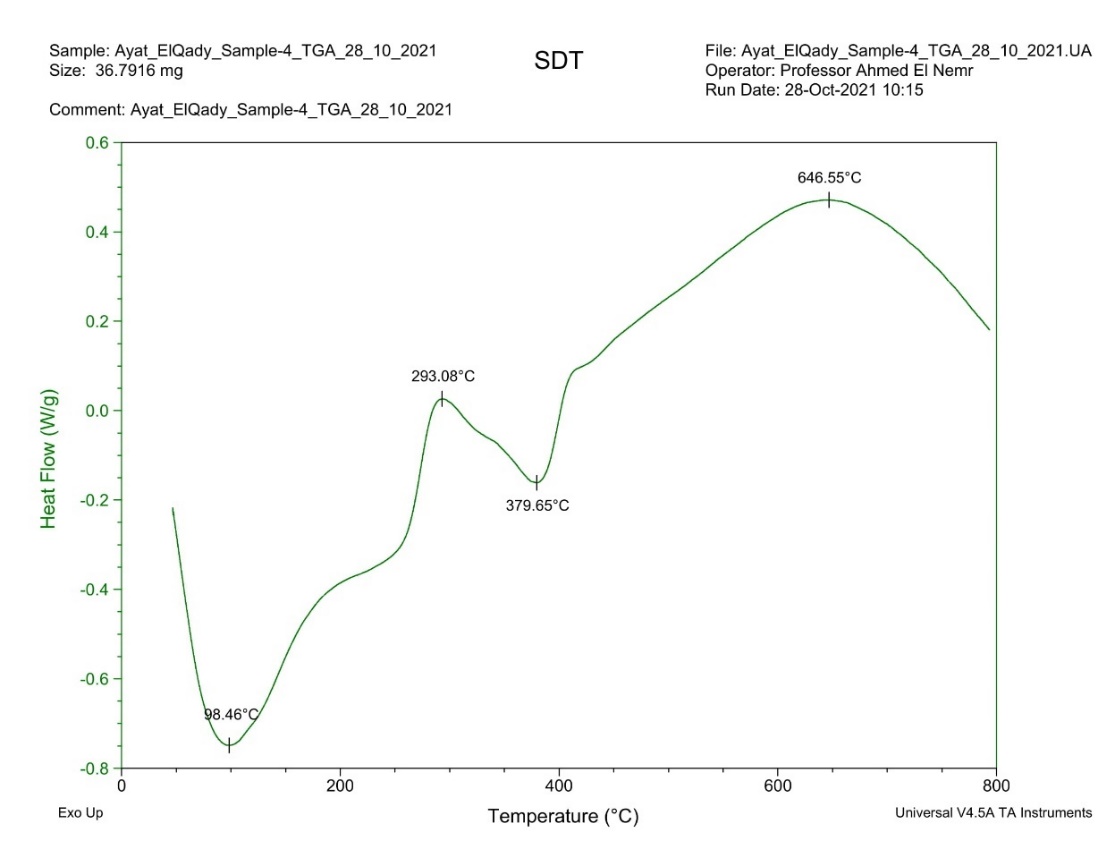
**

**Figure 15S:** DSC of formula; Chitosan (70%) / Starch (10%) / Castor (20%).

**
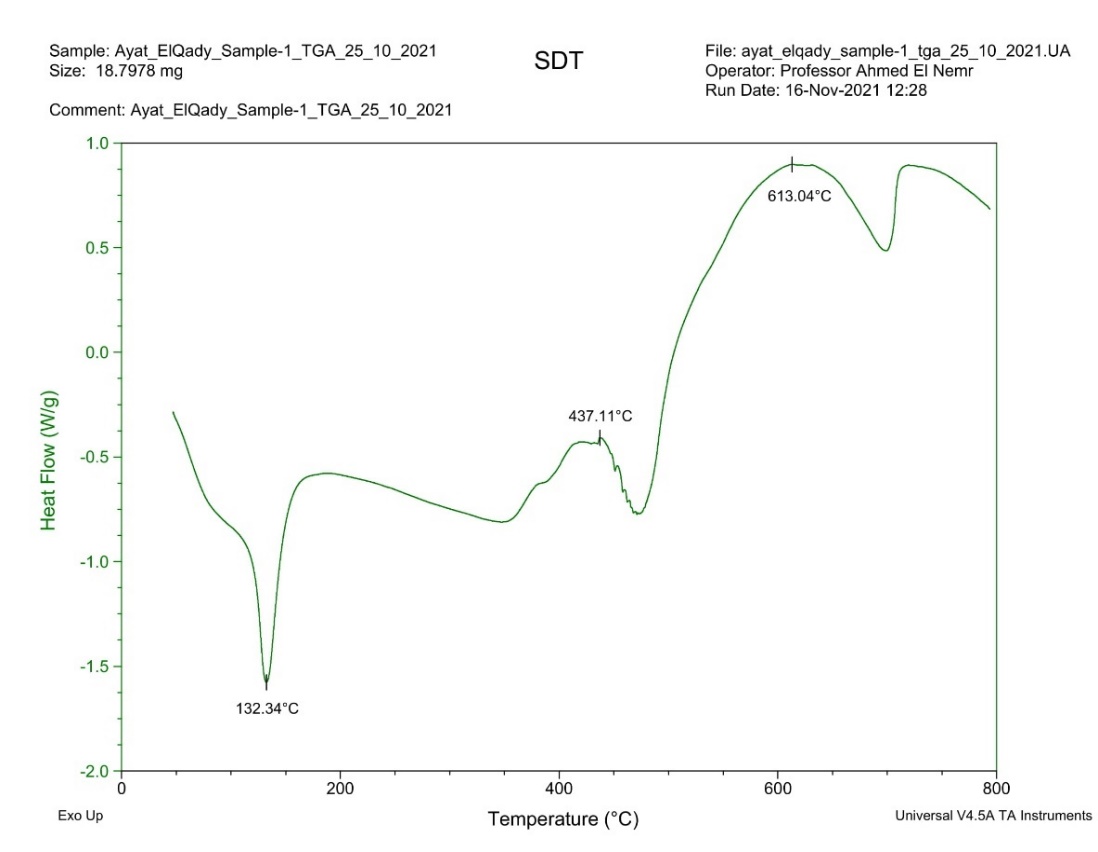
**

**Figure 16S:** DSC of polypropylene (PP) film.


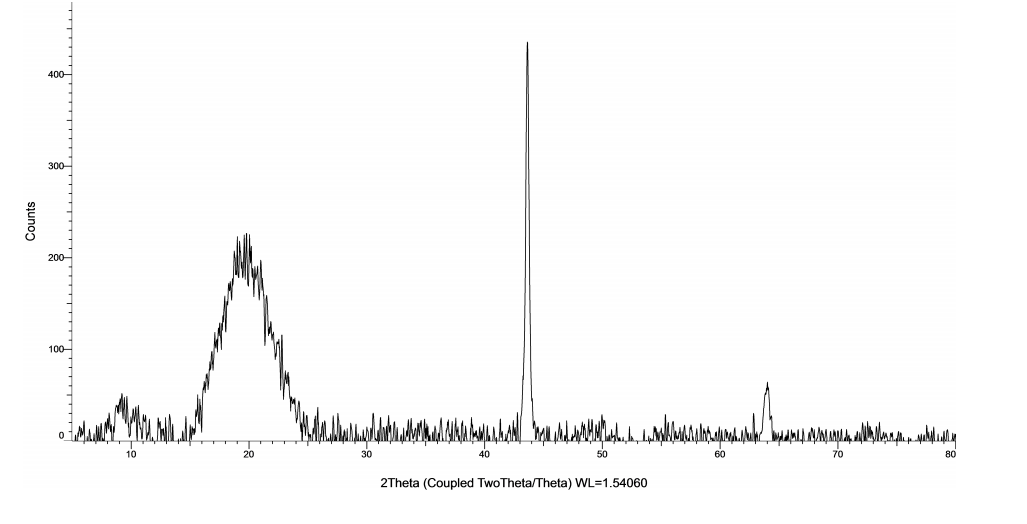


**Figure 17S:** XRD of extracted chitosan film.


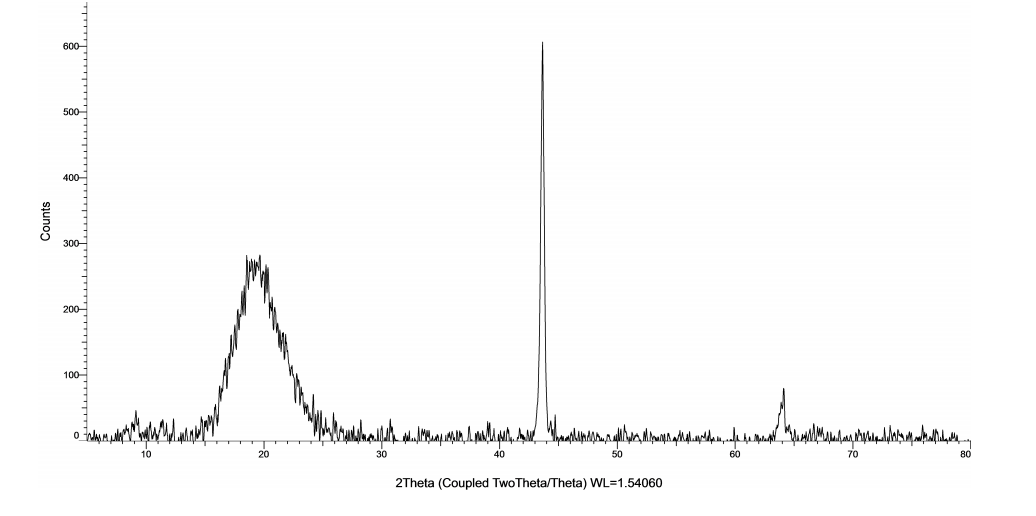


**Figure 18S:** XRD of formula; Chitosan (90%) / Starch (5%) / Castor (5%).


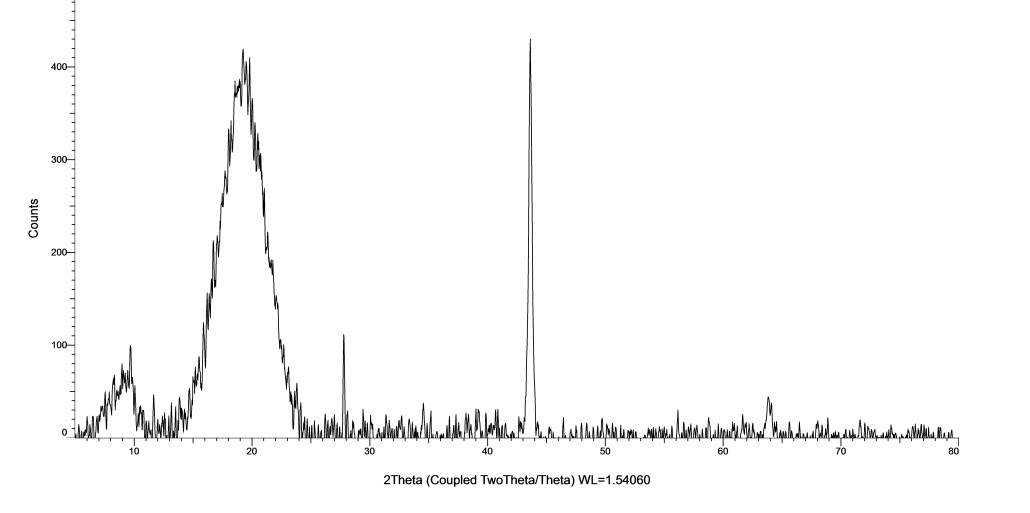


**Figure 19S:** XRD of formula; Chitosan (70%) / Starch (10%) / Castor (20%).


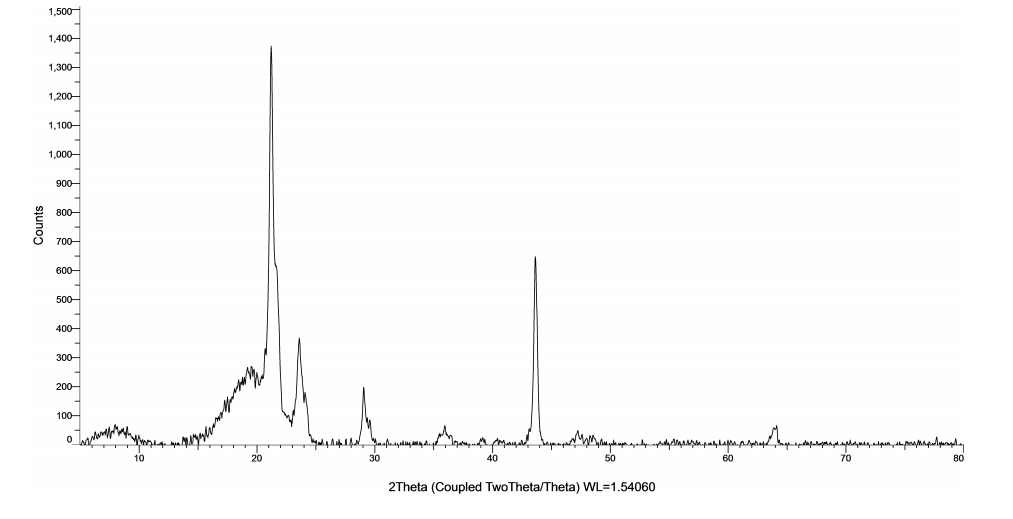


**Figure 20S:** XRD of polypropylene (PP) film.


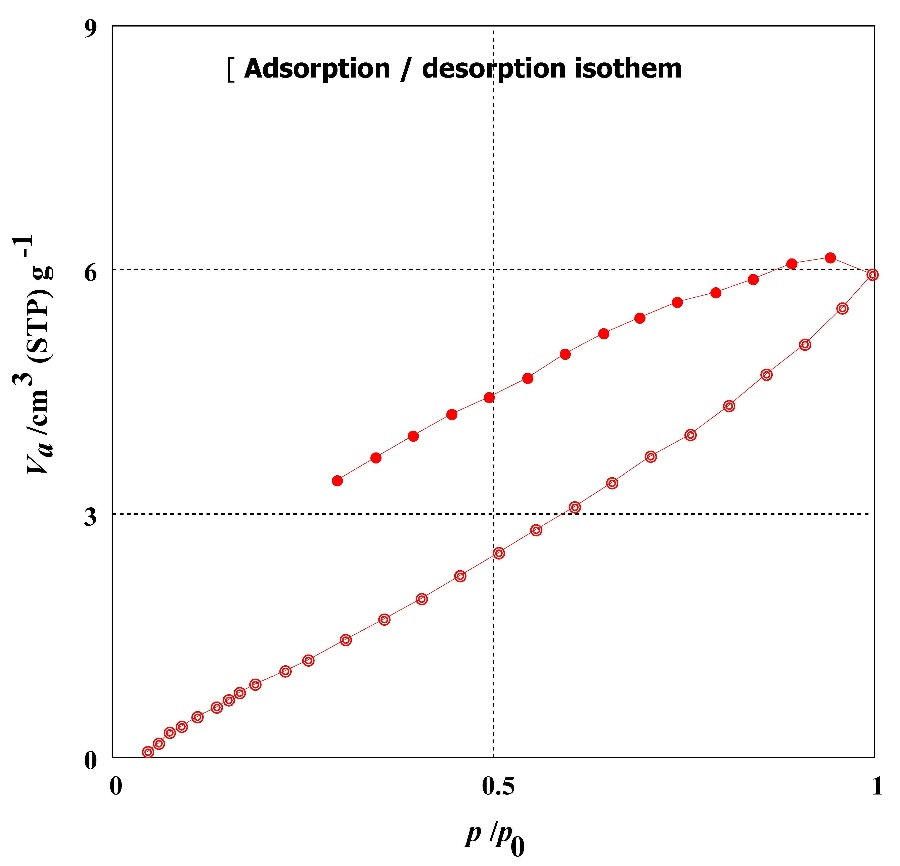


**Figure 21S:** Adsorption-desorption isotherm of Chitosan (70%) / Starch (10%) / Castor (20%).


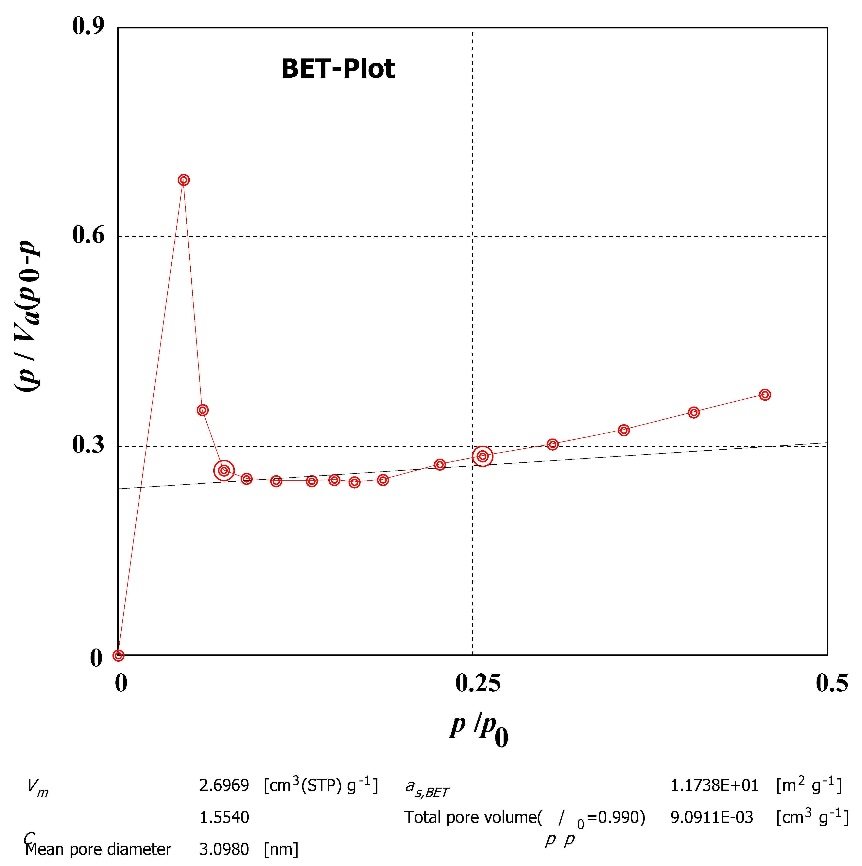


**Figure 22S:** BET analysis of Chitosan (70%) / Starch (10%) / Castor (20%).


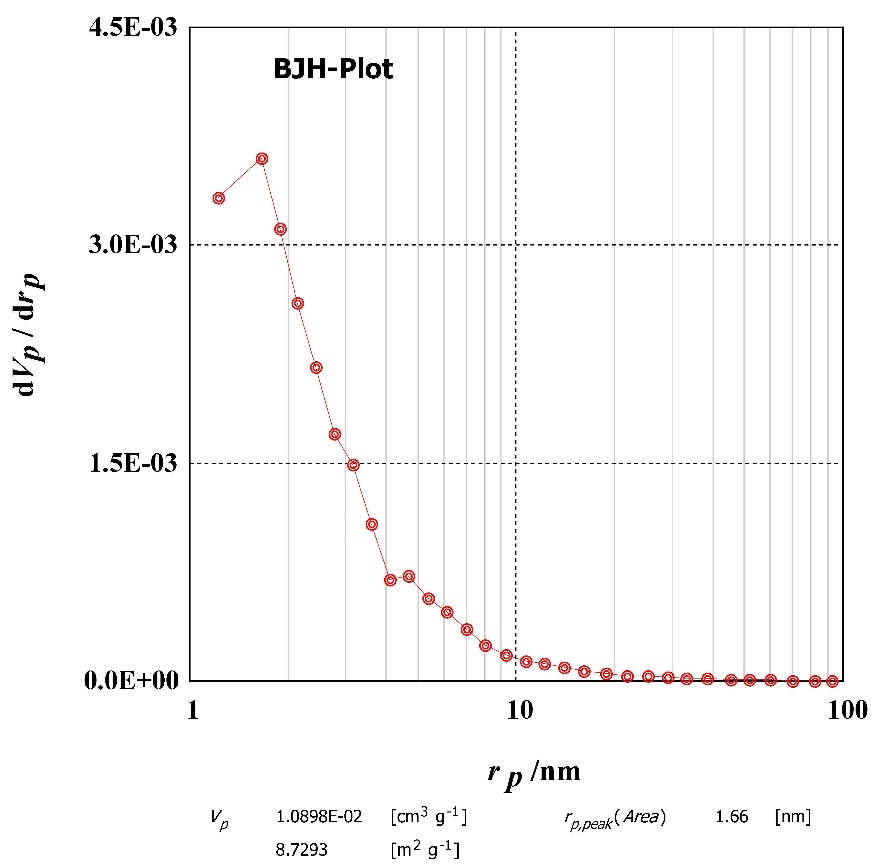


**Figure 23S:** BJH analysis of Chitosan (70%) / Starch (10%) / Castor (20%).
